# A Triple Threat Against Ovarian Cancer: Os(II)‐Pt(IV)‐Ceritinib Conjugates for Photodynamic Therapy, Chemotherapy, and Immunogenic Cell Death Induction

**DOI:** 10.1002/anie.202518623

**Published:** 2025-10-05

**Authors:** Marta Redrado, Sourav Acharya, Pierre Mesdom, Tomer Babu, James W. Southwell, Laiane S. Oliveira, Samia Hidalgo, Philippe Arnoux, Céline Frochot, Dan Gibson, Gilles Gasser

**Affiliations:** ^1^ Chimie ParisTech Institute of Chemistry for Life and Health Sciences, Laboratory for Inorganic Chemical Biology, PSL University, CNRS Paris 75005 France; ^2^ Institute for Drug Research, School of Pharmacy The Hebrew University of Jerusalem Jerusalem 91120 Israel; ^3^ Institut de Physique du Globe de Paris, Biogéochimie à l'Anthropocène des Eléments et Contaminants Emergents Paris 75005 France; ^4^ Université de Lorraine CNRS, LRGP Nancy F‐54000 France

**Keywords:** Bioinorganic chemistry, Medicinal inorganic chemistry, Metals in medicine: osmium, Photodynamic therapy, Platinum prodrugs

## Abstract

Metal‐based anticancer agents offer unique opportunities to integrate multiple therapeutic modalities within a single molecular framework. Herein, we present the first Osmium(II)‐Platinum(IV)‐Ceritinib conjugate (**Os‐Pt‐Cer**) that synergistically combines photodynamic therapy (PDT), chemotherapy, and immunotherapy via immunogenic cell death (ICD) induction. This heterobimetallic complex features a photoactive osmium(II) polypyridyl core, a platinum(IV) prodrug derived from oxaliplatin, and the kinase inhibitor ceritinib as an axial ligand. Upon deep‐red irradiation (*λ* = 740 nm), the conjugate exhibits potent antiproliferative activity in ovarian 2D cancer cells and 3D tumour spheroids, with IC_50_ values in the low nanomolar range. In addition, **Os‐Pt‐Cer** effectively inhibits cancer cell migration. Mechanistic studies reveal that the conjugate induces hallmarks of ICD, including calreticulin exposure, ATP release, HMGB1 secretion and phagocytosis. This multifunctional approach highlights the potential of osmium(II)‐platinum(IV) conjugates as promising candidates for overcoming resistance and immune evasion in high‐grade ovarian carcinoma.

## Graphical abstract

A novel Os(II)‐Pt(IV)‐Ceritinib (**Os‐Pt‐Cer**) conjugate enables triple‐action anticancer therapy by integrating photodynamic therapy, chemotherapy, and immunogenic cell death (ICD) induction into a single molecule. Upon near‐infrared light activation, this heterobimetallic complex exhibits potent antiproliferative effects in ovarian cancer models, triggering key ICD hallmarks, including calreticulin exposure, ATP release, and HMGB1 secretion.

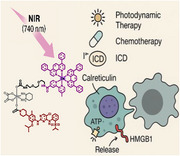

**Abbreviations**
ATP: adenosine triphosphate
Ce6: Chlorin e6
CRT: Calreticulin
DAMPs: Damage‐associated molecular patterns
DAPI: 4′,6‐diamidino‐2‐phenylindole
DIP: 4,7‐diphenyl‐1,10 phenanthroline
DSC: *N,N′*‐disuccinimidyl carbonate
ER: Endoplasmic Reticulum
ESI‐HRMS: Electrospray ionisation high‐resolution mass spectrometry
FDA: Food and Drug Administration
GSH: Glutathione
HGSOC: High‐Grade Serous Ovarian Carcinoma
HMB1: High‐Mobility Group Box 1
HPLC: High‐performance liquid chromatography
IC_50_
: Half maximal inhibitory concentration
ICP‐MS: Inductively Coupled Plasma Mass Spectrometry
ICD: Immunogenic cell death
IL: Intraligand transitions
LLCT: Ligand to ligand charge transfer
MCTS: Multicellular tumour spheroid
MLCT: Metal to ligand charge transfer
MTG: MitoTracker Green
NIR: Near‐infrared region
NSCLC: Non–small cell lung cancer
PDT: Photodynamic therapy
PpIX: Protoporphyrin
PS: Photosensitiser
ROS: Reactive oxygen species
UV: Ultraviolet

## Introduction

Cancer remains a leading cause of death worldwide, with ovarian cancer being among the most lethal.^[^ ^1^ ^]^ Cytoreduction and the use of platinum‐based drugs are the first line of treatment for this type of cancer.^[^ ^2^, ^3^ ^]^ Unfortunately, resistance and severe side effects limit their efficacy.^[^ ^4^ ^]^ Despite the promising results of non‐platinum agents such as ruthenium(II) or gold(I) complexes, platinum compounds remain the most effective treatment for ovarian cancer.^[^ ^5^, ^6^, ^7^, ^8^ ^]^ Pt(IV) prodrugs have emerged as a promising alternative, offering reduced systemic toxicity and enhanced selectivity through axial functionalisation with targeting or therapeutic moieties.^[^ ^9^ ^]^ In addition, the two axial positions constitute a potential opportunity for attaching active components, or luminescent dyes, that help to improve the therapeutic properties or imaging of the drugs, respectively. These ligands can be released when the Pt(IV) centre is reduced inside cells by cysteines in proteins rather than by small‐molecule reducing agents.^[^ ^10^, ^11^ ^]^

**Figure 1:**
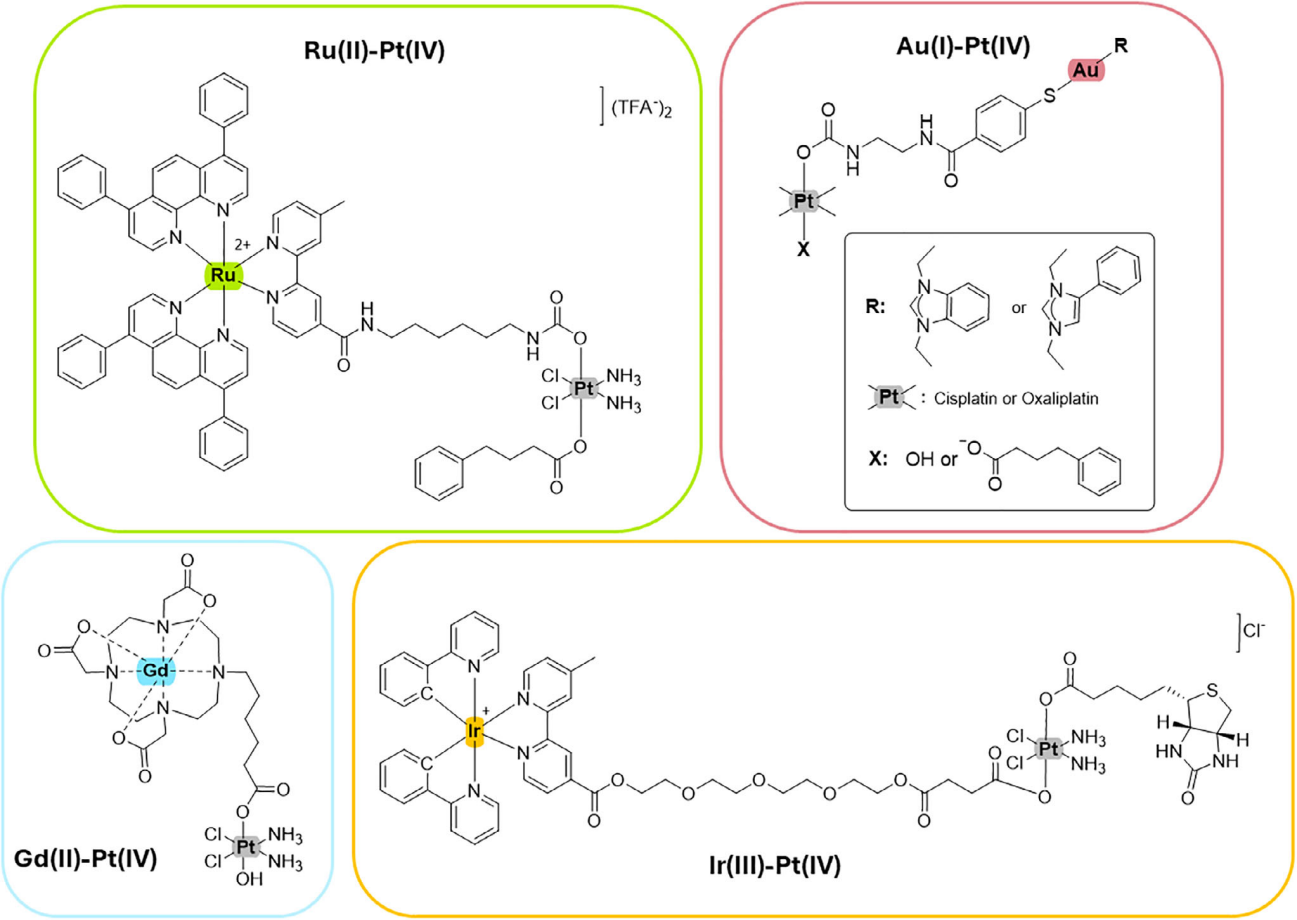
Chemical structures of reported heterometallic Pt(IV) conjugates: Ru(II),^[^ ^12^ ^]^ Au(I),^[^ ^13^ ^]^ Gd(III),^[^ ^14^ ^]^ or Ir(III).^[^ ^15^ ^]^

**Figure 2:**
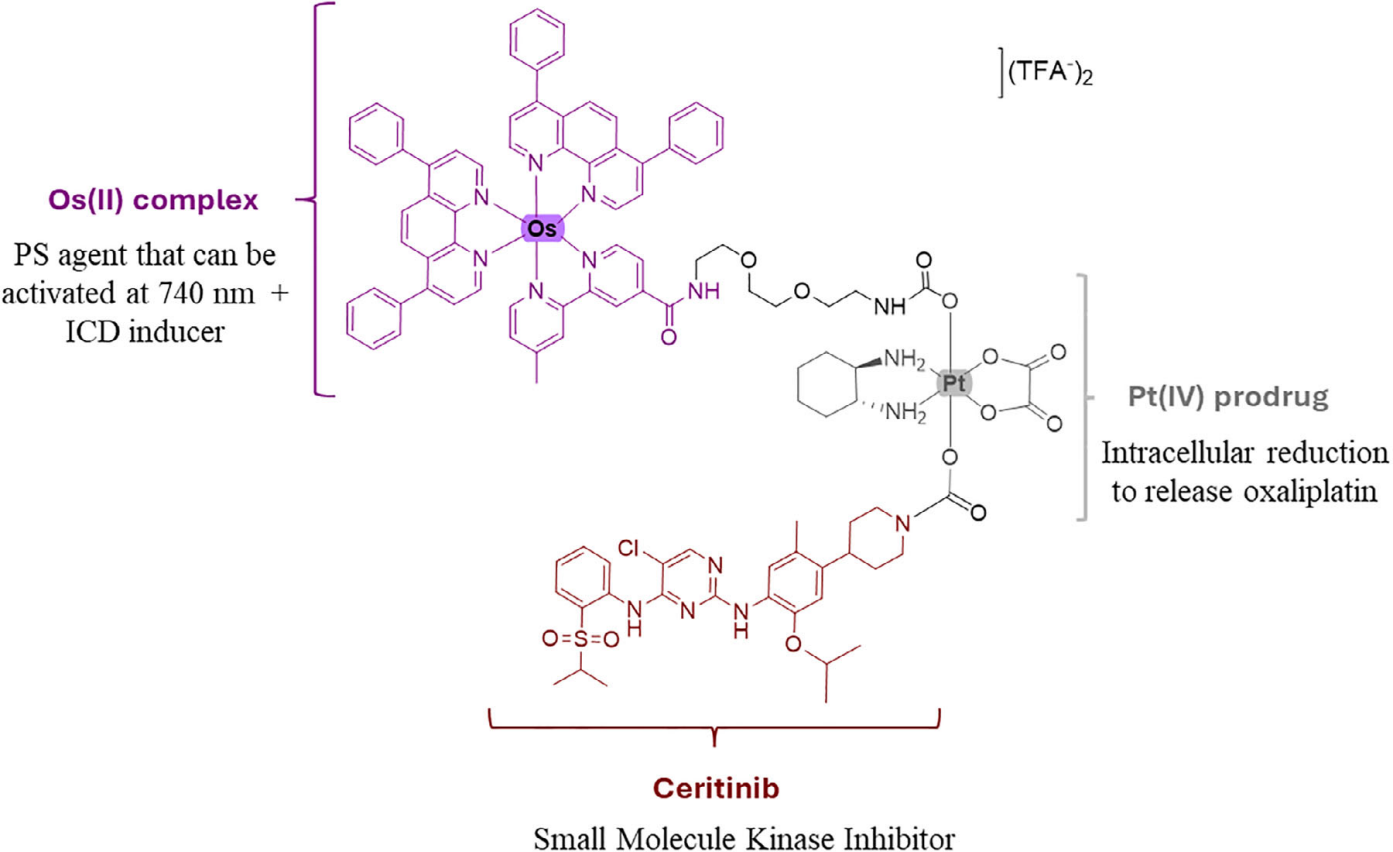
Depiction of the heterobimetallic Os(II)‐Pt(IV)‐Ceritinib (**Os‐Pt‐Cer**) conjugate as a triple‐action agent for ovarian cancer treatment. Pt(IV) centre is reduced inside the cells to oxaliplatin (an ICD inducer), releasing the Os(II) (PS and ICD inducer), and ceritinib from its axial sites.

Recent advances highlight the potential of combining Pt(IV) with other metal complexes to enhance therapeutic effects such as Ru(II),^[^ ^12^ ^]^ Au(I),^[^ ^13^ ^]^ Gd(III),^[^ ^14^ ^]^ or Ir(III),^[^ ^15^ ^]^ leading to outstanding antiproliferative activities (Figure 1). In this work, we introduce an innovative Os(II)‐Pt(IV)‐Ceritinib conjugate designed for dual photodynamic therapy (PDT) and chemotherapy. Os(II) polypyridyl complexes are recognised for their cytotoxicity and photosensitizing properties,^[^ ^16^ ^]^ while the kinase inhibitor ceritinib,^[^ ^17^ ^]^ disrupts mitochondrial respiration and induces the formation of reactive oxygen species (ROS). For this reason, we decided to include ceritinib as an additional axial ligand in the Pt(IV) complex, an oxaliplatin prodrug, Figure 2.

Beyond direct cytotoxicity, an emerging strategy in cancer therapy is the induction of immunogenic cell death (ICD), a regulated form of cell death that stimulates an immune response against cancer cells.^[^ ^18^ ^]^ ICD is characterised by endoplasmic reticulum (ER) stress, ROS generation, and autophagy, leading to the release of damage‐associated molecular patterns (DAMPs) among which calreticulin (CRT), ATP, and high mobility group box 1 (HMGB1) are ICD hallmarks that can enhance tumour recognition by the immune system.^[^ ^19^ ^]^ Metal complexes,^[^ ^20^ ^]^ and notably, *N*‐heterocyclic carbene gold complexes,^[^ ^21^, ^22^ ^]^ osmium and platinum‐based drugs have shown potential as ICD inducers, standing out as metal‐based agents with such demonstrated in vivo immunogenicity.^[^ ^23^ ^]^ Given that high‐grade serous ovarian carcinoma (HGSOC) is often characterised by immune evasion and poor response to conventional immunotherapies, strategies that elicit ICD could be particularly beneficial.^[^ ^24^ ^]^

Herein, we report the synthesis and investigation of an Os(II)‐Pt(IV)‐Ceritinib conjugate (**Os‐Pt‐Cer**), the first bimetallic Os‐Pt complex designed to integrate PDT, chemotherapy, and immunogenic cell death (ICD) induction, for potential application in ovarian cancer treatment. Particular efforts were devoted to elucidate its multimodal mechanism of action, aiming to establish a robust therapeutic strategy that enhances both cytotoxic efficacy and immune recognition.

## Results and Discussion

### Synthesis and Characterisation

**Scheme 1:**
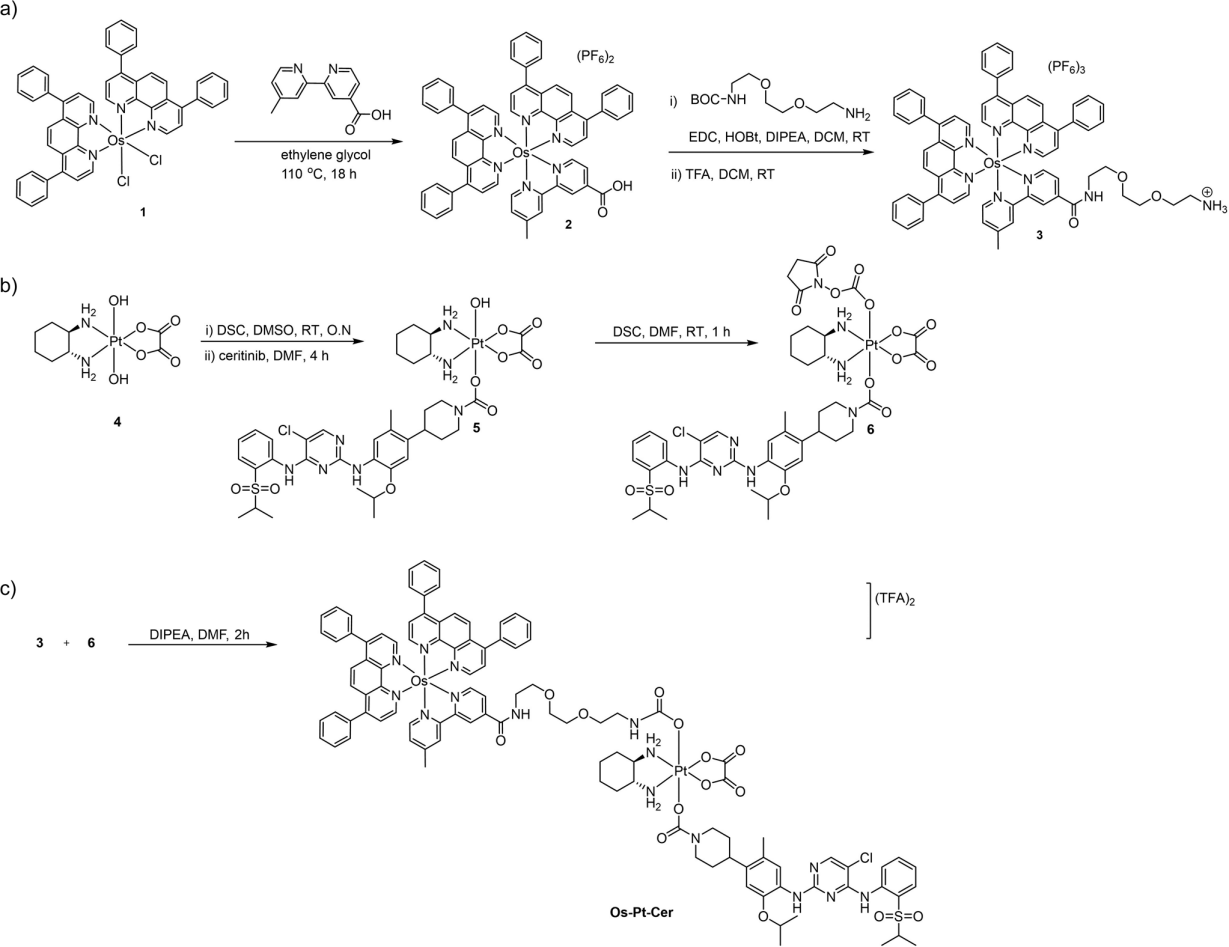
Synthesis of **Os‐Pt‐Cer**; a) the synthesis of the Os(II) complex that was functionalized with a primary amine linker; b) the preparation of the Pt(IV) derivative of oxaliplatin that was activated with DSC, c) the conjugation of the two halves to obtain the **Os‐Pt‐Cer**.

The synthetic strategy to develop the novel **Os‐Pt‐Cer** conjugate is depicted in Scheme 1. Briefly, the Os(II) complex **3** and the *N,N′*‐disuccinimidyl carbonate (DSC) activated Pt(IV)‐Ceritinib complex **6** were prepared and purified as detailed in the Supporting Information. The target **Os‐Pt‐Cer** conjugate was obtained by the nucleophilic attack of the primary amine of **3** on the electrophilic carbonyl carbon of **6** to form a stable carbamate linkage. The **Os‐Pt‐Cer** complex was purified by HPLC (>99% purity) and characterized by ESI‐HRMS.

**Figure 3:**
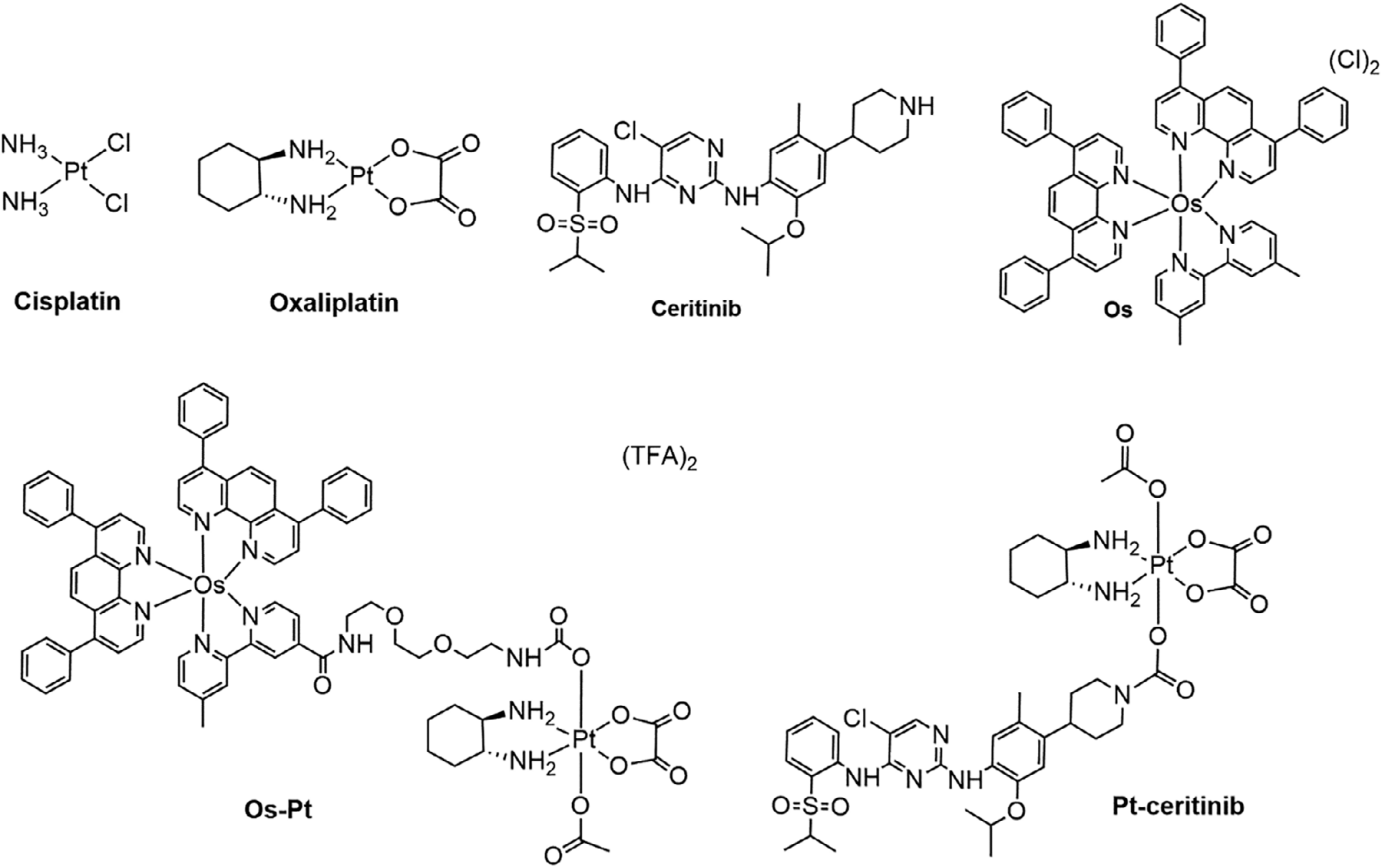
The chemical structures of the control compounds used in this study.

As controls, we used commercial oxaliplatin, cisplatin, and ceritinib, and prepared the Os(II) complex (**Os**), and two dual‐action prodrugs; the osmium‐platinum complex (**Os‐Pt**) and the platinum‐ceritinib complex (**Pt‐Ceritinib**) depicted in Figure 3.

### Photophysical Studies

The optical properties of all compounds were investigated in an aerated acetonitrile solution at room temperature where the most relevant data is shown in Table 1. The UV–vis spectra of the different complexes allow us to determine whether they absorb in the desired phototherapeutic window (i.e., 600–850 nm).^[^ ^25^ ^]^ **Os‐Pt‐Cer**, **Os‐Pt,** and **Os** show similar absorption and emission spectra, exhibiting three absorption bands, as is commonly observed for similar osmium complexes.^[^ ^5^, ^26^ ^]^ An intense peak at 280 nm, which is assigned to the intra‐ligand (IL) π→π* transitions of DIP (4,7‐diphenyl‐1,10 phenanthroline), two broad peaks (with maxima at ca. 445 and 500 nm, respectively) attributed to the Metal‐to‐Ligand‐Charge‐Transfer (MLCT) Os(dπ)→ ligand(π*), and finally a broadband covering the region 570–750 nm, attributed to spin‐forbidden MLCT transitions, generated by the strong spin‐orbit coupling of the osmium centre, see Figure S17. The emission spectra of these complexes displayed structureless emission bands with maximum peaks at 730–745 nm, Figure S18. These emission bands can be attributed mainly to ^3^MLCT and ^3^LLCT transitions, as reported for similar complexes.^[^ ^27^ ^]^ In addition, excited‐state lifetimes, photoluminescence quantum yields, and singlet‐oxygen quantum yields were measured, using [Ru(bpy)_3_] as the reference standard; the corresponding values are summarized in Tables S2 and S3.

**Table 1:** Photophysical data of studied compounds measured in an aerated CH_3_CN solution.

	UV–vis × 10^3^ (ε [dm^3^ mol^−1^ cm^−1^])	λ_em_ (λ_ex_) (nm)
**Os‐Pt‐Cer**	290 (26.2); 445 (6.3); 500 (5.4); 590 (1.6)	740 (475)
**Os‐Pt**	290 (9.5); 445 (4.7); 500 (4.2); 590 (0.1)	745 (475)
**Os**	290 (44.0); 445 (15.7); 500 (14.1); 590 (4.9)	730 (475)
**Pt‐Cer**	305 (10.5)	–
**Pt(OAc)*_2_***	285 (10.0)	–
**Ceritinib**	305 (4.0)	–

### Stability and Reduction

The stability of the **Os‐Pt‐Cer** complex in the dark was monitored by HPLC in 10% DMSO in RPMI medium containing serum. It was found to be stable, with a half‐life (*t*_1/2_) of 5 days (Figure S20). To evaluate whether the complex can release the free drugs upon reduction, we performed reduction studies using an excess of ascorbate. The progress of the reaction was monitored by HPLC, which confirmed the release of **3** and ceritinib in their free form after reduction (Figure S21). The photostability of complexes **Os‐Pt‐Cer**, **Os‐Pt,** and **Os** were investigated under different irradiation conditions. These complexes were dissolved in the biological medium RPMI 1640 at a concentration of 10 µM. Thereafter, they were irradiated for one hour using the same conditions as used during phototoxicity assays (670 nm (13.50 J cm^−2^) or 740 nm (12.60 J cm^−2^)). The stability of these complexes was analysed by UV–vis spectroscopy after irradiation (Figure S19). No significant differences were observed, attesting to good stability in cell culture medium.

### Cytotoxicity and Phototoxicity

The 2D cytotoxicity and phototoxicity profiles of **Os‐Pt‐Cer** and controls, were evaluated in a panel of cancerous cell lines, including A2780 (ovarian carcinoma), A549 (non‐small cell lung carcinoma), and MCA205 (murine fibrosarcoma), as well as a non‐cancerous retinal pigment epithelial cell line (RPE‐1). Cells were treated with the compounds (0.003–100 µM) for 4 h, either kept in the dark or irradiated at 670 nm (13.5 J cm^−^^2^) (Table S4 and Figure S22) or 740 nm (12.6 J cm^−^^2^) and viability was assessed 44 h post‐treatment. To verify robustness, the incubation was extended to 72 h. The IC_50_ values obtained after prolonged exposure did not differ significantly from those measured at 48 h, suggesting that the maximal cytotoxic effect of **Os‐Pt‐Cer** is reached within this timeframe.

In the absence of light, **Os**, **Os‐Pt**, and **Os‐Pt‐Cer** have low to moderate activity against A2780 ovarian cancer cells and are essentially inactive against MCA205, A549 and the non‐cancerous RPE‐1, making them good potential candidates for phototherapy (Table 2). The **Pt‐Cer** complex lacks activity in all cell lines. The full dose–response curves and statistical analyses, are provided in the (Figure S22–S27). Cisplatin, PpIX (Protoporphyrin) and Ce6 (Chlorin e6), well‐known PSs, were used as additional controls.^[^ ^28^, ^29^ ^]^

**Table 2:** IC_50_ (µM) values in normoxic conditions, in the dark and upon irradiation at 740 nm (60 min, 12.60 J·cm^−2^) on A2780, RPE‐1, MCA205, and A549 cells following a 4 h exposure of the cells to the compounds and a total of 48 h of incubation. Average of three independent measurements.

A2780				RPE‐1		
Compound	Dark	Irrad. 740 nm	PI	Dark	Irrad. 740 nm	PI
*Os‐Pt‐Ceritinib*	9.40 ± 0.06	0.42 ± 0.04	24	14.5 ± 0.3	1.73 ± 0.2	8
*Os‐Pt*	11.50 ± 0.1	0.39 ± 0.03	27	>100	1.57 ± 0.1	≥63
*Os*	39.24 ± 0.2	0.29 ± 0.1	135	>100	0.86 ± 0.2	≥116
*Pt‐Ceritinib*	>100	–	–	>100	–	–
*Ceritinib*	2.61 ± 0.5	–	–	4.76 ± 0.2	–	–
*Oxaliplatin*	46.15 ± 0.2	–	–	>100	–	–
*PPIX*	>100	17.3 ± 0.01	≥6	>100	41.6 ± 0.3	≥2
*Cisplatin*	1.92 ± 0.4	–	–	15.2 ± 0.7	–	–

MCA205				A549		
Compound	Dark	Irrad. 740 nm	PI	Dark	Irrad. 740 nm	PI
*Os‐Pt‐Ceritinib*	>100	3.21 ± 0.67	≥31	>100	5.89 ± 0.91	≥17
*Os‐Pt*	>100	1.68 ± 0.22	≥60	>100	12.6 ± 0.12	≥8
*Os*	42.31 ± 1.1	0.64 ± 0.04	66	>100	1.67 ± 0.67	≥58
*Pt‐Ceritinib*	73.8 ± 0.86	–	–	35.2 ± 0.13	–	–
*Ceritinib*	6.97 ± 0.70	–	–	3.80 ± 0.09	–	–
*Oxaliplatin*	0.80 ± 0.09	–	–	2.80 ± 0.13	–	–
*Ce6*	>100	1.88 ± 0.20	≥53	>100	5.86 ± 0.82	≥17
*Cisplatin*	0.72 ± 0.20	–	–	1.11 ± 0.33	–	–

In all three cancer cell lines, as well as in the non‐cancerous RPE‐1, irradiation at 740 nm significantly enhanced cytotoxicity compared with the experiments performed in the absence of light. In all tested cell lines, following irradiation, **Os** has the lowest IC_50_ values (0.29–1.67 µM) while **Os‐Pt‐Cer** has somewhat higher IC_50_ values. The PI values (IC_50_ dark/ IC_50_ irradiation 740 nm) of **Os** are significantly higher than those of **Os‐Pt‐Cer**. Among the tested lines, A2780 ovarian cancer cells exhibited the highest sensitivity to **Os‐Pt‐Cer**, **Os‐Pt**, and **Os** (Table 2). Under irradiation at 740 nm, **Os‐Pt‐Cer** exhibited a 4‐fold tumour selectivity (IC_50_ non‐cancerous RPE‐1/IC_50_ cancer cell) only against the A2780 cells, but not against MCA205 and A549.

The Os(II)‐based PS allows deep‐tissue activation in the deep‐red spectral region (670–740 nm), enabling efficient light‐triggered toxicity even under low‐energy conditions. The phototherapeutic response in A2780 cells highlights **Os‐Pt‐Cer** as a potent and multimodal anticancer agent. Its performance under clinically‐relevant irradiation conditions makes it a promising candidate for light‐activated therapy in solid tumours.

The promising results obtained with **Os** and **Pt‐Os‐Cer** in the A2780 cancer cell line prompted us to investigate their cytotoxic and phototoxic activity in a multicellular tumour spheroid (MCTS) model in comparison to ceritinib, oxaliplatin and cisplatin.^[^ ^30^ ^]^ These 3D spheroids are used as organotypic models of solid tumours, simulating key tumour characteristics such as hypoxia, growth patterns, and metabolism, thereby enabling a more accurate estimation of the in vivo antitumour activity compared with the 2D models.^[^ ^31^ ^]^

**Figure 4:**
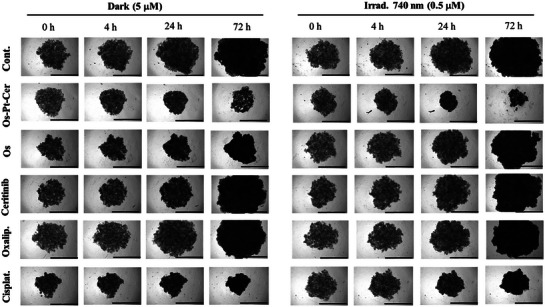
Changes in the growth kinetics of A2780 MCTSs treated with complex **Os‐Pt‐Cer**, **Os**, ceritinib, oxaliplatin, and cisplatin at 0.5 and 5 µM for irradiated and dark groups, respectively, and non‐treated as control. Images were collected on 0, 4, 24, and 72 h after the creation of the groups. Scale bar: 1000 µm.

A2780 spheroids (ca. 500 µm in diameter) were treated with increasing concentrations of the compounds and after 24 h of incubation, the medium was replaced with fresh medium, and the cells were either kept in the dark or irradiated for 1 h at 740 nm. Images of the spheroids were taken every 24 h for 3 days. Remarkably, following irradiation, A2780 MCTSs treated with **Os‐Pt‐Cer** at 0.5 µM had a significantly reduced diameter after 72 h, while the diameters of its components, **Os**, oxaliplatin and ceritinib increased with time (Figure 4). Interestingly, even in the dark, incubation with **Os‐Pt‐Cer** resulted in the reduction of the diameter attesting to the existence of effective non‐phototoxic mechanisms for cytotoxicity.

Additionally, the phototoxicity was tested via a fluorometric resazurin assay in A2780 MCTSs (Table 3). **Os‐Pt‐Cer** exhibited high cytotoxicity toward A2780 MCTSs under light irradiation conditions (IC_50, 740nm_: 0.2 ± 0.2 µM). (Figure S29). Notably, discrepancies between growth kinetics and resazurin‐based IC_50_ values were observed for some compounds. This can be attributed to the different biological endpoints and time frames of the assays: spheroid diameter was monitored over 72 h, whereas resazurin measurements were performed after 7 days, potentially capturing delayed cytotoxic effects due to differences in drug cellular uptake.

**Table 3:** IC_50_ (µM) values in normoxic conditions, in the dark, and upon irradiation at 740 nm (60 min, 12.60 J·cm^−2^) on A2780 MCTSs after one week of incubation.

Compound	Dark	Irrad. 740 nm	PI
**Os‐Pt‐Cer**	>5	0.2 ± 0.2	≥26
**Os**	>5	>5	≥1
**Ceritinib**	>5	–	–
**Oxaliplatin**	0.5 ± 0.7	–	–
**Ce6**	>5	0.7 ± 0.4	≥7
**Cisplatin**	2.0 ± 0.4	–	–

**Figure 5:**
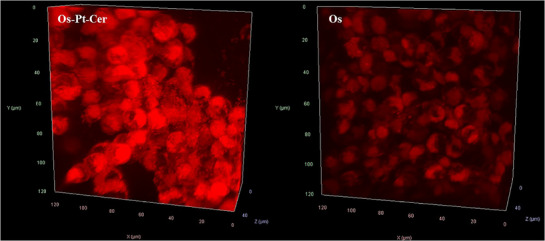
Confocal microscopy images of A2780 multilayer incubated with **Os‐Pt‐Cer** and **Os** complexes in the dark for 24 h at the same concentrations (2 µM).

To better understand the exceptional behaviour of **Os‐Pt‐Cer** in the A2780 multilayer system, we investigated its penetration capability using fluorescence confocal microscopy. The results were compared with those obtained for **Os** to assess the differences in cellular uptake and distribution. As shown in Figure 5, **Os‐Pt‐Cer** penetrates this MCTS very efficiently, explaining its exceptional cytotoxicity in 3D models.

### Cellular uptake and Localization Studies

**Figure 6:**
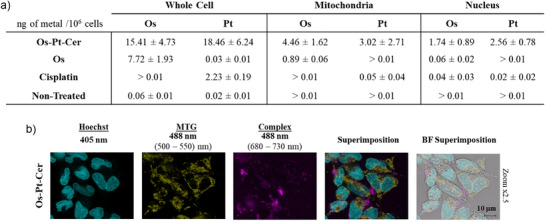
Uptake studies. a) Os and Pt content (ng per million cell) in A2780 cells (5 µM) after 4 h of incubation with **Os‐Pt‐Cer** and **Os** and cisplatin complexes, and non‐treated cells; b) Confocal microscopy images of A2780 cells incubated with **Os‐Pt‐Cer** complex for 4 h, followed by the addition of organelle‐selective trackers MitoTracker Green (30 min incubation, 100 nM) and Hoechst (30 min incubation, 1 µg mL^−1^).

Following this, the uptake mechanism of the **Os‐Pt‐Cer** conjugate was studied by inductively coupled plasma mass spectrometry (ICP‐MS) to evaluate its cellular targets in A2780 cell line.^[^ ^32^ ^]^ As shown in Figure 6a, the conjugate **Os‐Pt‐Cer** and **Os** show similar osmium distribution inside cells after 4 h of incubation. However, the osmium content of cells treated with **Os‐Pt‐Cer** conjugate is twice as high. The localization of the released Os(II) complex and Pt(II) complex (oxaliplatin) were investigated by extracting the nucleus and mitochondria, followed by osmium and platinum quantification by ICP‐MS. Low levels of both osmium and platinum were detected in the mitochondria and nucleus, suggesting that the majority of both metals remain distributed in the cytoplasm or other cell compartments. Notably, despite this reduction process, no major divergence in the subcellular localization of Os(II) and Pt(II) was observed, indicating that both metal fragments may follow similar trafficking routes during early cellular processing. Cisplatin, **Os** and non‐treated cells were used as controls.

To confirm the localization results obtained by ICP‐MS, a fluorescence confocal microscopy assay was performed. The intrinsic emission of Os(II) complexes was used to determine its cellular localization. MitoTracker Green (MTG) and Hoechst 33 342 were employed to specifically dye mitochondria and the nucleus, respectively. As shown in Figure 6b, **Os‐Pt‐Cer** was internalised into A2780 cells after only 4 h of incubation. **Os‐Pt‐Cer** (magenta) and MTG (yellow) staining patterns were clearly distinct, indicating that osmium did not accumulate in either mitochondria or the nucleus (blue). This finding was further supported by colocalization analysis using the Coloc2 plugin in ImageJ, which yielded Pearson colocalization coefficients of ‐0.22 with MTG and ‐0.30 with Hoechst, confirming minimal overlap between the osmium content of **Os‐Pt‐Cer** and these cellular organelles.

### Scratch Assay

Ceritinib has been investigated in combination with other drugs, such as Dasatinib, for its capacity to limit cell migration.^[^ ^33^, ^34^ ^]^ The high cytotoxicity of **Pt‐Os‐Cer** against A2780 cells (Table 2) led us to explore whether the incorporation of ceritinib enhances the conjugate's antimetastatic activity. To evaluate the in vitro cell migration of **Os‐Pt‐Cer**, a scratch assay was performed to mimic the migration of cells in vivo.^[^ ^35^ ^]^ As additional controls, **Os‐Pt** and **Pt‐Cer** were selected, and compared with free oxaliplatin, ceritinib, and the **Os** complex, and non‐treated cells.

**Figure 7:**
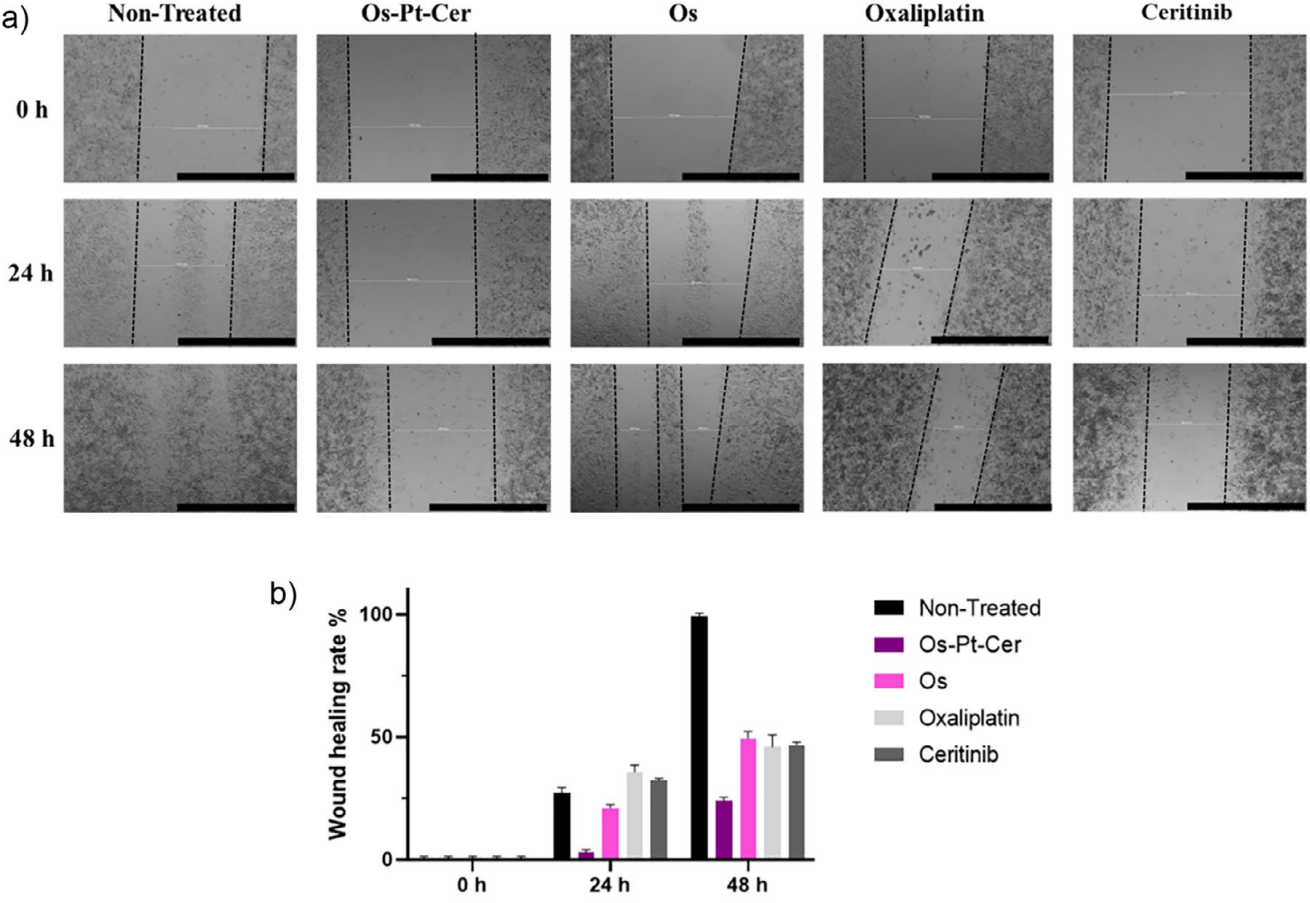
a) Migration images of A2780 cells after 0, 24, and 48 h of treatment with IC_20_ concentrations of **Os‐Pt‐Cer**, **Os**, oxaliplatin, and ceritinib, respectively, or non‐treated cells. Black lines indicate the dimensions of the scratches. The images are representative of one successive experiment. Scale bar: 1000 µm. b) Comparative graph of healing percentage over studied time.

A2780 cells were seeded at 90% of confluency on a 12‐well plate. Once the monolayer was formed, a scratch was introduced using a cell scraper. The selected compounds were added at their respective IC_20_ concentrations (i.e., the concentration of the drug inhibiting 20% of the cell viability), and the scratch was monitored at different incubation times (0, 24, and 48 h) to check the differences in healing between treated and non‐treated cells. As shown in Figure 7, **Os‐Pt‐Cer** effectively inhibited the migration and invasion of A2780 cells at the IC_20_ concentration after 48 h of treatment. When compared with the controls, these results demonstrate that the inclusion of ceritinib in the conjugate mitigates the migration of A2780 cells, as it does and is known to do for the ceritinib control.

### Immunogenic Cell Death Induction

Triggering the immune system to recognise and attack tumour cells is an important approach in cancer therapy. A key mechanism behind this is ICD, which stimulates the immune system by releasing so‐called “danger signals”, also known as DAMPs from dying tumour cells. The most well‐known hallmarks of ICD are the translocation of CRT to the cell membrane, the release of ATP, and the release HMGB1,^[^ ^19^ ^]^ which are essential for attracting immune cells and activating an anti‐tumour immune response.^[^ ^36^ ^]^

To evaluate whether **Os‐Pt‐Cer** is an ICD inducer, we treated A2780 ovarian, and MCA205 cells under both dark and light (740 nm) conditions and assessed the three major hallmarks. **Os**, Ce6, oxaliplatin, cisplatin, and non‐treated cells were evaluated as controls.

**Figure 8:**
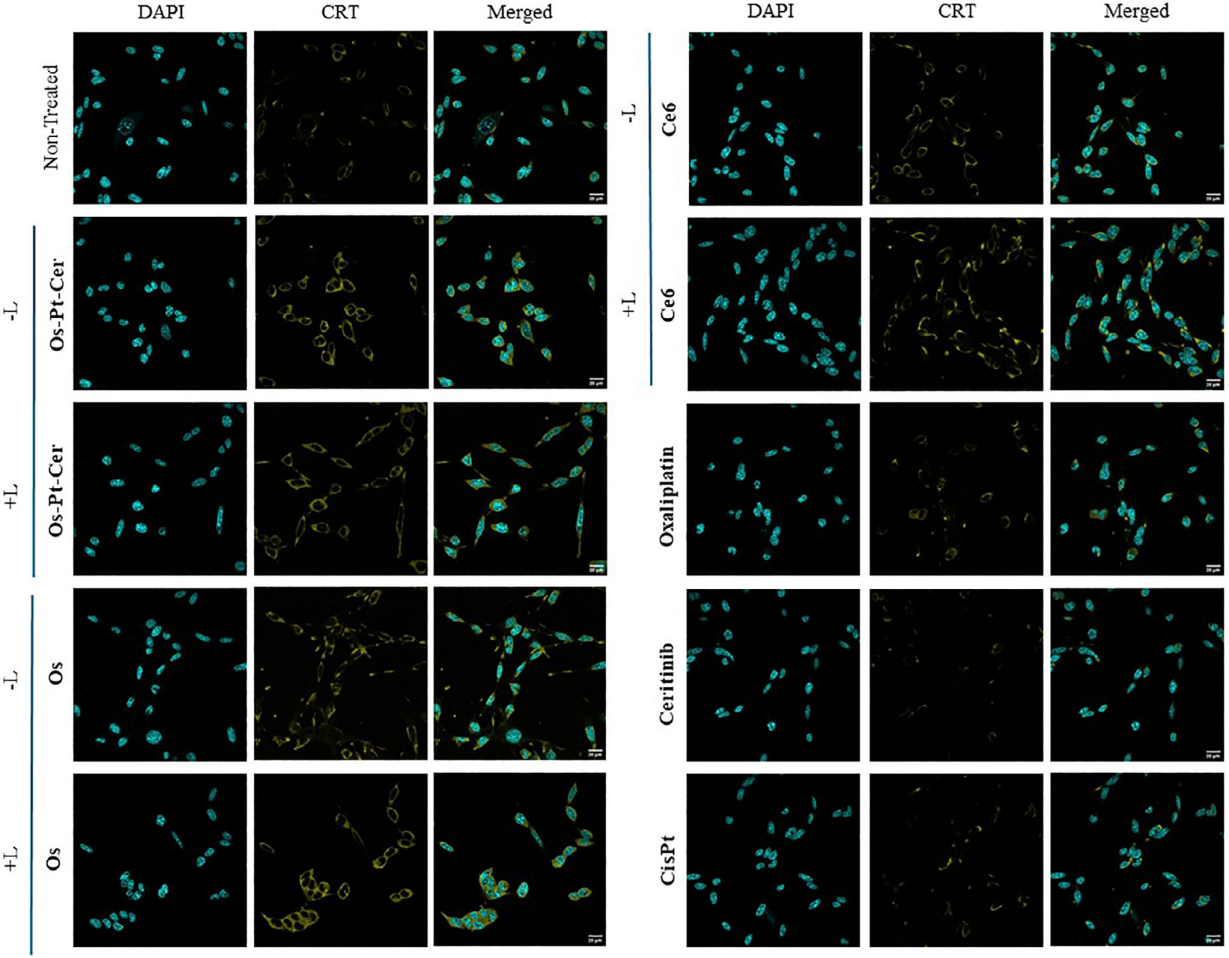
Translocation and changes in calreticulin (CRT) exposure on MCA205 cells determined by confocal fluorescence microscopy after 4 h treatment under dark (−L) and light (+L) (740 nm, 1 h) conditions, and an additional 16 h of incubation. DAPI: Nuclear fluorescence dye. Scale bar: 20 µm.

Surface exposure of CRT was measured via confocal fluorescence microscopy after staining with an anti‐CRT antibody (ab2907). Cells treated with **Os‐Pt‐Cer** showed increased exposure of CRT compared to non‐treated cells in both studied conditions (Figures 8 and S32). The surface exposure in the dark might be due to the oxaliplatin, a bona fide ICD inducer. Specifically, CRT expression increased by 67% upon irradiation and 63% in the dark, compared with non‐treated cells. Notably, the effect was stronger than that observed for oxaliplatin (CRT increase = 21%) used as a positive control for ICD induction, or **Os** complex (57% under irradiation, and 48% in the dark). These results indicate that **Os‐Pt‐Cer** can induce ICD exposition even in dark conditions, that can be attributed to the presence of **Os** and oxaliplatin in its structure.

**Figure 9:**
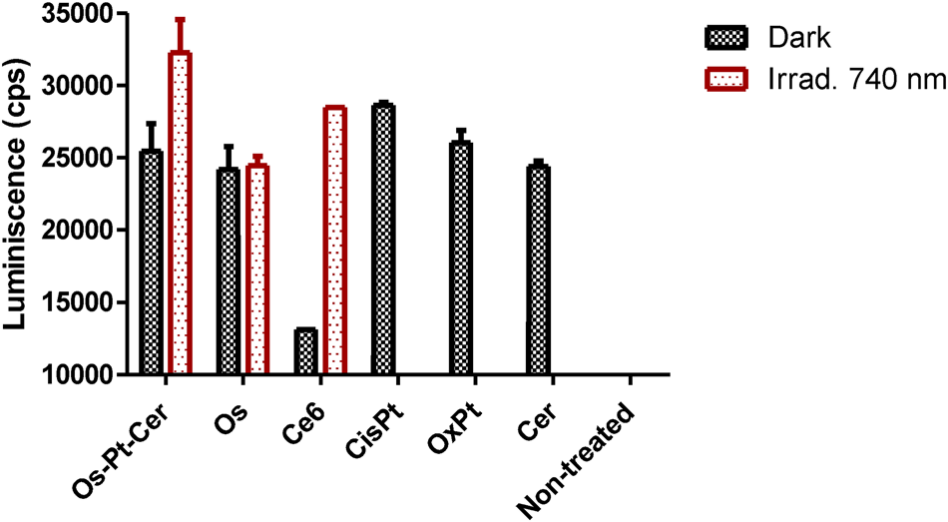
Quantification of extracellular ATP levels using a luciferase‐based assay following 4 h incubation in MCA205 cells.

ATP release into the extracellular medium was quantified using a luminescent ATP Detection Assay Kit (ab113849). As shown in Figure 9, treatment with **Os‐Pt‐Cer** led to an increase in ATP release under light irradiation as well as in the dark compared to control cells. Again, this response was comparable or superior to Oxaliplatin, supporting the capacity of the conjugate to initiate ICD.

**Figure 10:**
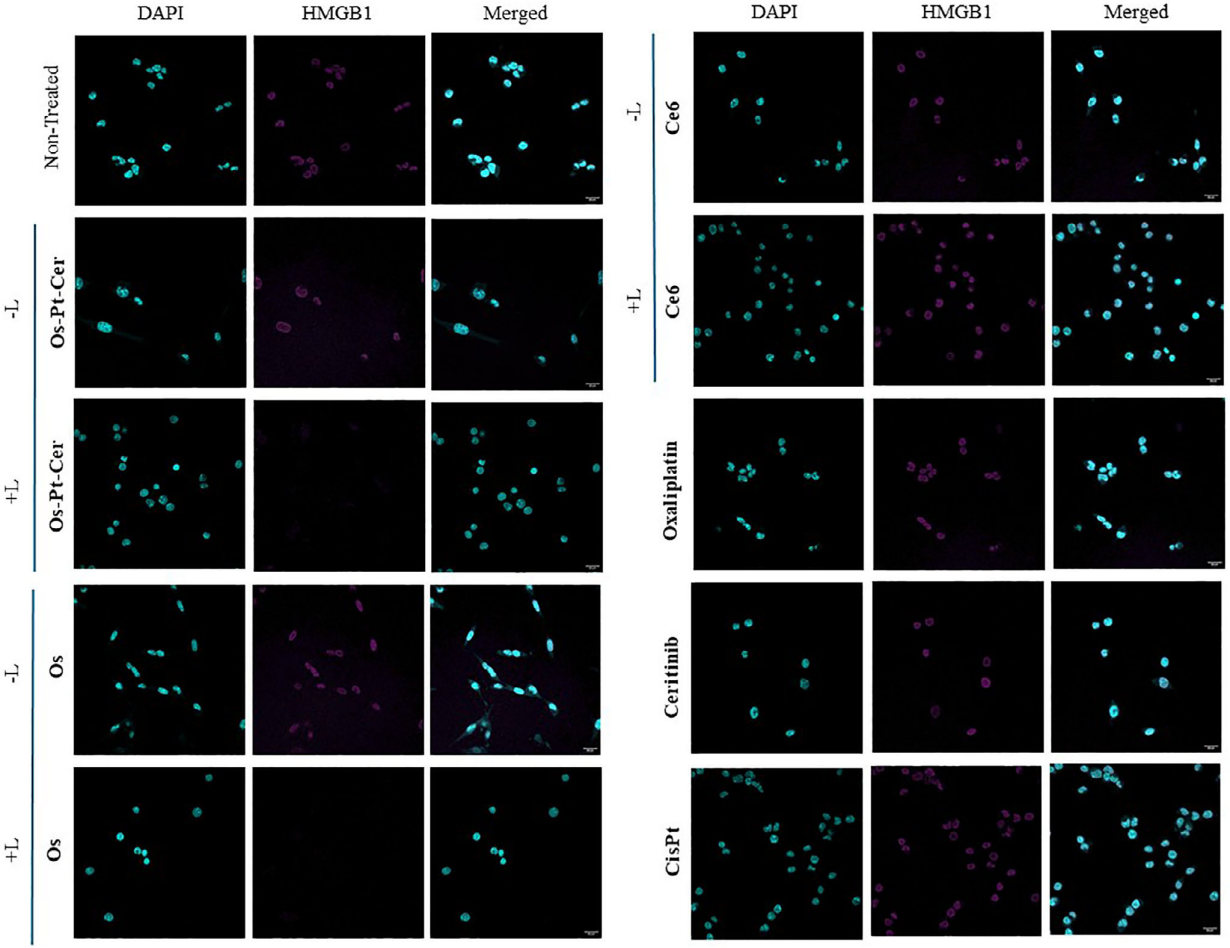
Release of HMGB1 measured by confocal microscopy in MCA205 cells after 4 h of incubation under dark (−L) and light (+L) (740 nm, 1 h) conditions. Scale bar: 20 µm.

Finally, HMGB1 release was analysed by confocal microscopy after 4 h of treatment. Upon irradiation, **Os‐Pt‐Cer** triggered a total secretion of HMGB1 while the non‐irradiated condition showed a weaker response, Figures 10 and S34. These results align with those in CRT assays. Taken together, these results demonstrate that **Os‐Pt‐Cer** is a potent ICD inducer, particularly under light activation (740 nm), but also in the dark, positioning it as a strong candidate for combination therapies involving immune system modulators or vaccine strategies.

Since the **Os‐Pt‐Cer** induces the ICD hallmark in MCA205 and A2780 cells (Figure S34), we further investigated whether the treated tumour cells could be effectively recognised and engulfed by macrophages. Phagocytosis is a key cleaning mechanism in ICD. It is primarily mediated by macrophages, which are responsible for clearing dying or stressed tumour cells,^[^ ^37^ ^]^ once CRT, an endoplasmic reticulum chaperone, is translocated to the plasma membrane, acting as an “eat‐me” signal for macrophages.^[^ ^38^ ^]^

MCA205 cells were treated with **Os‐Pt‐Cer,** as well as with their respective controls, at their 48 h IC_50_ and IC_25_ concentrations. All compounds were incubated for 4 h with the cells, which were either irradiated at 740 nm (1 h) or kept in the dark. 16 h post‐treatment, cancer cells were stained with CellMask Green, while RAW 264.7 macrophages were stained with CellMask DeepRed. Co‐culture was performed at a 1 : 2 (macrophage : tumour cells) ratio for 3 h. Thereafter, the phagocytosis was evaluated by flow cytometry and confocal microscopy.

**Figure 11:**
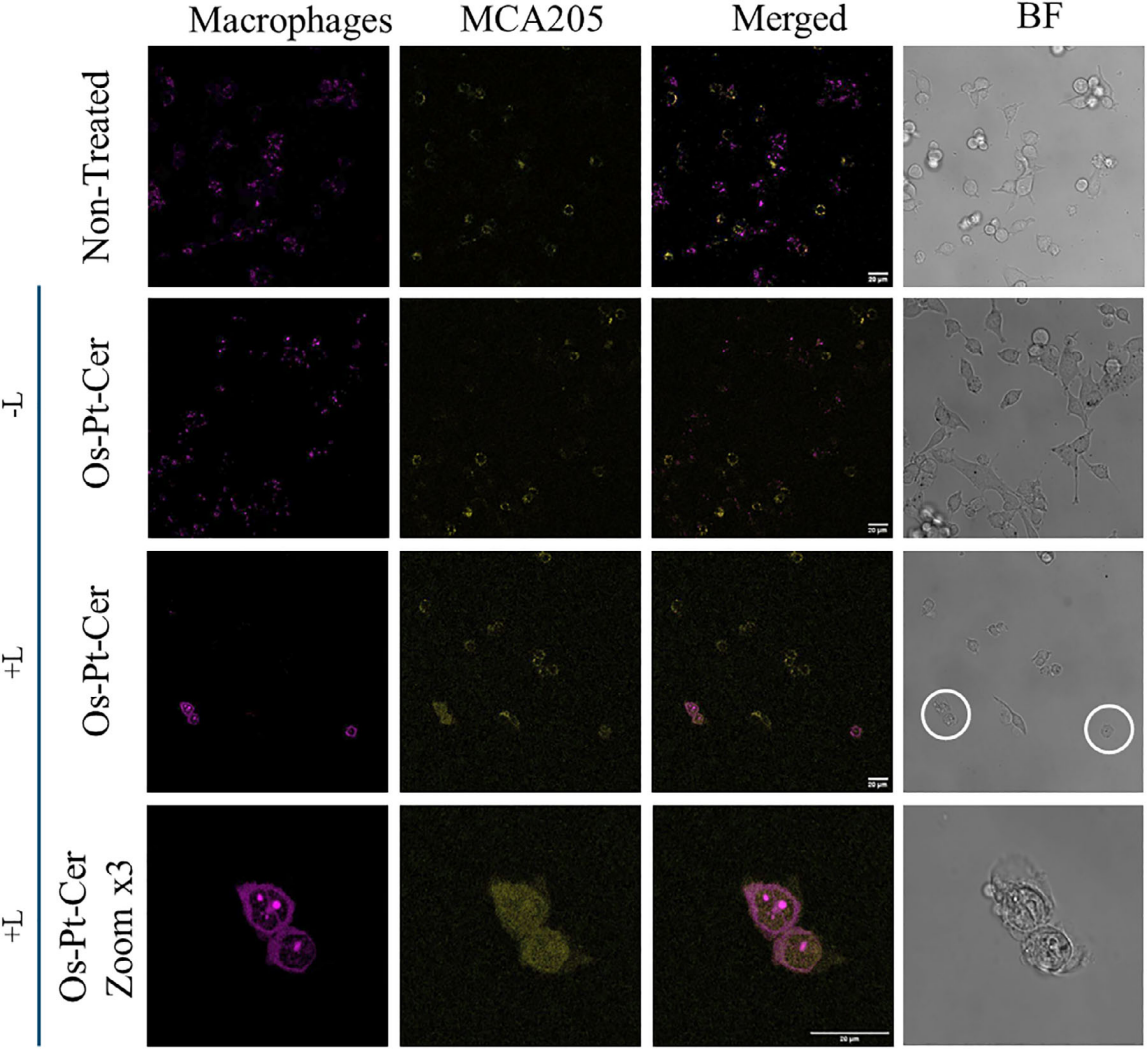
Confocal microscopy images show phagocytosis of MCA205 cells after being treated with **Os‐Pt‐Cer** after 4 h of incubation, half of the samples were kept in dark (−L) or were irradiated (+L) (740 nm, 1 h). After 16 h, they were co‐incubated with RAW 264.7 macrophages for 3 h. MCA205 were labelled with CellMask Green™ at 405 nm (500–550 nm), and RAW 264.7 with CellMask DeepRed™ at 520 nm (650–750 nm).

As shown in Figure 11, **Os‐Pt‐Cer** effectively promotes phagocytosis of treated cancer cells upon light irradiation. This response is consistent with the translocation of CRT to the plasmic membrane, ultimately facilitating the immune‐mediated clearance of cancer cells.

**Figure 12:**
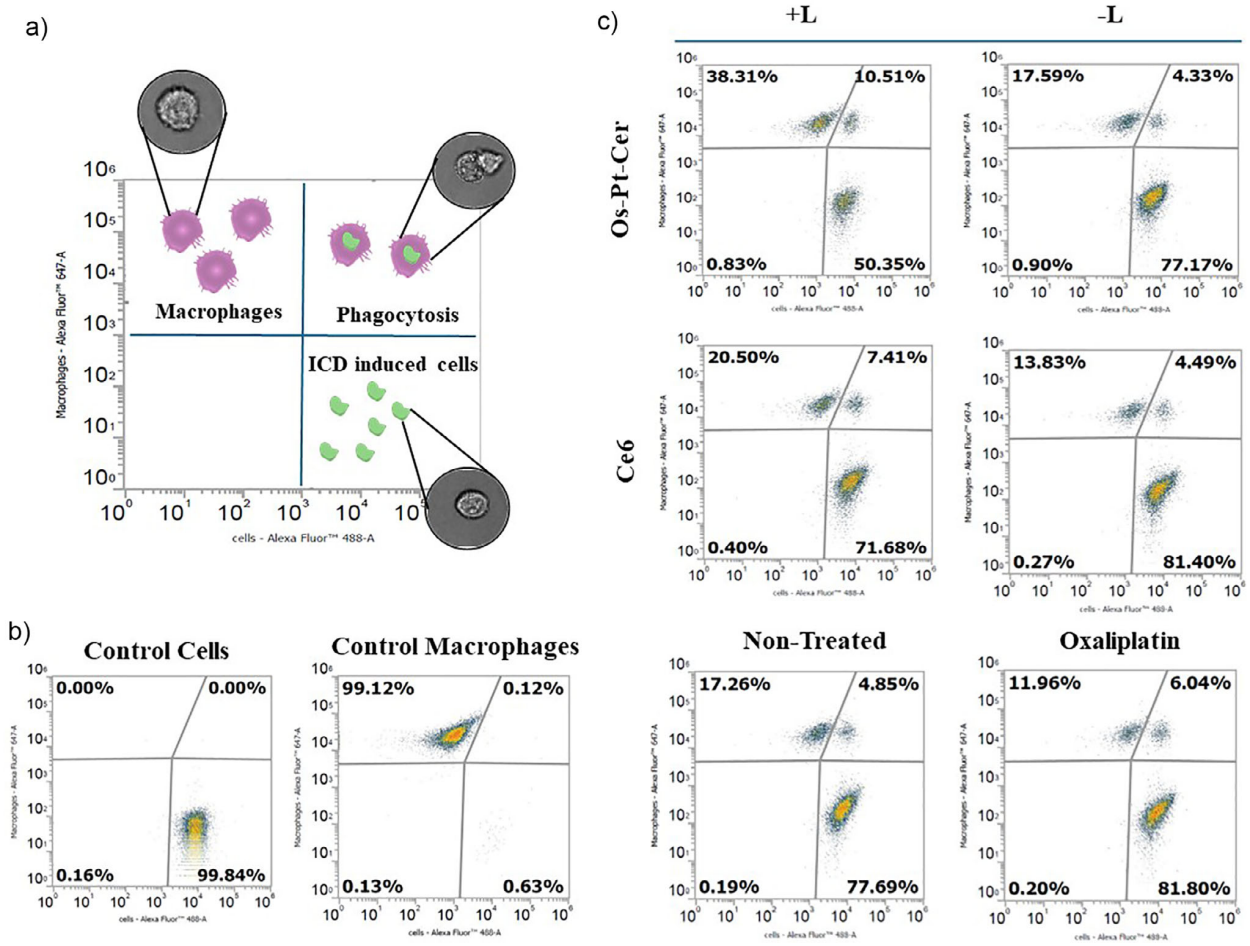
Flow cytometry phagocytosis study. a) Schematic representation of the plots with real images of cells obtained by flow cytometry; b) Dot plots of the controls: MCA205 cells were stained with CellMask™ Green, and RAW 264.7 macrophages with CellMask™ Deep Red; c) Flow cytometric analysis of phagocytosis after 3 h of coincubation. The samples were kept in dark (−L) or were irradiated (+L) (740 nm, 1 h). Dot plots of non‐treated cells, and treated with ICD inducers as Ce6 and Oxaliplatin are shown as additional controls. Concentration IC_50,_ 48 h values.

To quantify this phagocytic response, a flow cytometry assay was performed, see Figure 12. MCA205 cells were incubated with **Os‐Pt‐Cer** for 4 h, after which half of the samples were either kept in the dark (−L) or irradiated (+L) for 1 h (740 nm). After an additional 16 h, the cells were co‐incubated with RAW 264.7 macrophages for 3 h. Prior to co‐incubation, MCA205 cells were stained with CellMask Green, and RAW 264.7 macrophages with CellMask Deep Red. Flow cytometry analysis was used to quantify phagocytosis, identifying double‐positive (green–red) events as phagocytic macrophages. Notably, **Os‐Pt‐Cer** promoted this immunogenic clearance more efficiently under irradiation (10.5 %) compared to established ICD inducers such as Ce6 (7.4 %) and oxaliplatin (6.0 %). These findings further confirm the immunogenic nature of the cell death triggered by **Os‐Pt‐Cer** and underscore its potential as a dual photodynamic–chemotherapeutic agent capable of engaging both direct cytotoxicity and immune‐mediated tumour clearance.

## Conclusions

In this study, we present a novel conjugate (**Os‐Pt‐Cer**) that combines a dual‐action Pt(IV) complex with oxaliplatin and ceritinib as chemotherapy agents, with an Os(II) complex as a photosensitiser, which is activated at 740 nm. The conjugate enters cancer cells, where the Pt(IV) centre undergoes reduction to Pt(II), releasing its axial ligands. This conjugate exhibits phototoxicity in the low nanomolar range, as observed in both 2D monolayer ovarian cancer cells and 3D spheroids. Remarkably, the conjugate also displays antimetastatic activity and is a highly efficient inducer of ICD, as evidenced by the presence of the DAMPs, and importantly, confirmed by phagocytosis assays. We strongly believe that this innovative conjugate and more generally, the strategy of combining two chemotherapeutic agents with distinct mechanisms of action, with a photosensitiser for PDT, holds significant promise for future research in cancer therapy.

## Author Contributions

The manuscript was written through the contributions of all authors. All authors have approved the final version of the manuscript.

## Conflict of Interests

The authors declare no conflict of interest.

## Associated Content

The data that support the findings of this study are available in the Supporting Information, which includes general measurement conditions, analysis instrumentation, experimental procedures and characterization, photophysical studies, and in vitro assays.

## Supporting information



Supporting Information

## Data Availability

The data that support the findings of this study are available in the supplementary material of this article.
