# Supplementary material for: A Triple Threat Against Ovarian Cancer: Os(II)‐Pt(IV)‐Ceritinib Conjugates for Photodynamic Therapy, Chemotherapy, and Immunogenic Cell Death Induction
Source: Angew Chem Int Ed Engl. 2025 Oct 5;64(49):e202518623. doi: 10.1002/anie.202518623 (PMC12668304; doi:10.1002/anie.202518623)
Supplement: Supplementary file 1 — Supporting Information [file ANIE-64-e202518623-s001.pdf]

Electronic supplementary information (ESI)

A Triple Threat Against Ovarian Cancer: Os(II)-Pt(IV)-Ceritinib Conjugates for Photodynamic Therapy, Chemotherapy, and Immunogenic Cell Death Induction

Dr. Marta Redrado,[a,#] Dr. Sourav Acharya,[b,#] Dr. Pierre Mesdom,[a] Dr. Tomer Babu,[b] Dr. James W. Southwell,[a] Dr. Laiane S. Oliveira,[a] Samia Hidalgo,[c] Dr. Philippe Arnoux,[d] Dr. Céline Frochot,[d] Prof. Dr. Dan Gibson*,[b] and Prof. Dr. Gilles Gasser,*,[a]

[a] Chimie ParisTech, PSL University, CNRS, Institute of Chemistry for Life and Health Sciences, Laboratory for Inorganic Chemical Biology, 75005 Paris, France.

[b] Institute for Drug Research, School of Pharmacy, The Hebrew University of Jerusalem, 91120 Jerusalem, Israel.

[c] Institut de Physique du Globe de Paris, Biogéochimie à l'Anthropocène des Eléments et Contaminants Emergents, 75005 Paris, France.

[d] Université de Lorraine, CNRS, LRGP, F-54000 Nancy, France.

# These authors have contributed equally to the work.

Experimental Section

Materials and Methods **3**

Procedure and Characterization **8**

Spectroscopic measurements **20**

(Photo-)cytotoxicity on 2D cell monolayers **23**

(Photo-)cytotoxicity on 3D multicellular tumor spheroids (MCTS) **31**

Intracellular distribution by confocal fluorescence microscopy **32**

Scratch Assay **33**

Externalization of calreticulin **34**

Detection of extracellular HMGB1 protein **35**

Extracellular ATP release **36**

Materials and Methods

^{1}H, ^{13}C(1H), ^{31}P(1H), DEPT 135, HSQC, COSY, HMBC and NOESY NMR spectra were recorded on at room temperature (303 K) either on a Bruker Avance III HD 400 MHz spectrometer (equipped with a BBFO Plus Smartprobe, ^{1}H: 400 MHz ; ^{13}C: 100 MHz ; ^{31}P: 162 MHz), or on a Bruker Avance Neo 500 MHz spectrometer (equipped with a BBFO Smartprobe BB & ^{19}F/^{1}H, ^{1}H: 500 MHz ; ^{13}C: 125MHz; ^{31}P: 202 MHz). ^{31}P(^{1}H) NMR chemical shifts are reported in ppm and externally referenced to 85% H_3PO_4 at 0 ppm. Ile de France Region is gratefully acknowledged for financial support of 500 MHz NMR spectrometer of Chimie ParisTech in the framework of the SESAME equipment project (n°16016326). Multiplicity abbreviations are as follows; s for singlet, d for doublet, t for triplet, q for quartet qn for quintet, sx for sextet, sp for septet, dd for double doublet, td triple doublet, ddd for doublet of doublet of doublets, m for multiplet, br for broad and app for apparent. NMR assignments were aided by various combinations of DEPT 135, HSQC, COSY, HMBC and NOESY experiments, where required.Reaction mixtures were analyzed by thin layer chromatography using Merck silica gel 60F254 aluminums plates and visualized by UV light. Column chromatography was performed with Claricep Flash Silica Column (20 g) purchased from Phenomenex. All materials were obtained from commercial suppliers (Acros, Alfa-Aesar, Apollo Scientific, BLD Pharma, Fisher Scientific, Fluka, Sigma-Aldrich, Tokyo Chemical Industry) and used as supplied. The water used in the reactions was freshly distilled prior to use. Ceritinib (CAS No: 1032900-25-6) was purchased from 1PlusChem. The progress of the reactions for Pt(IV) complexes was monitored using an analytical HPLC system (Thermo Scientific UltiMate 3000) with a reverse-phase C18 column (Phenomenex Kinetex, 100 mm length, 4.60 mm internal diameter, 2.6 µm Particle size, 100 Å pore size). The purity and retention time (RT) of the synthesized compound reported here were measured using the same analytical HPLC system, with a 0.1% trifluoroacetic acid (TFA) in water and acetonitrile gradient at a flow rate of 1 mLmin^{-1}. Reaction mixtures were purified on a preparative HPLC system (Thermo Scientific UltimaMate 3000 station) equipped with a reverse-phase C18 column (Phenomenex Luna, 250 × 21.2 mm, 10 µm, 100 Å), using a similar type of mobile phase at a flow rate of 15 mLmin^{-1}. UV detection was set at 220 nm. The fractions were combined and lyophilized to get the pure compounds. The fractions were combined and lyophilized to get the pure compounds. The newly synthesized Pt(IV) complexes were characterized by ESI-MS and the purity (>95%) was determined by HPLC measurements.

HRMS

ESI-MS experiments were carried out using an LTQ-Orbitrap XL (Thermo Fisher Scientific, Courtaboeuf, France) operated in positive or negative ionization mode, with a spray voltage at 3.6 kV and an ion transfer capillary temperature of 275 °C. Sheath gas flow rate was set at 30. Applied voltages depended on the theoretical mass of the desired product within the sample (see Table S1) for the ion transfer capillary and the tube lens, respectively. Detection was achieved in the Orbitrap with a

resolution of 100,000 (at m/z 400) where the m/z range in profile mode depended (see Table S1) on the applied voltage. The spectra were recorded using the acquisition software XCalibur 2.1 (Thermo Fisher Scientific, Courtaboeuf, France). Typical automatic gain control (AGC) and maximum injection time were 2.10^5 ions and 500 ms. 1 µscan was acquired. Internal lockmass was enabled using in-house reference compounds. 1-10 µL (10 µg mL^{-1}) were injected using a Thermo Finnigan Surveyor HPLC system (Thermo Fisher Scientific, Courtaboeuf, France) with a continuous infusion of methanol at 60 µL min^{-1}.

Table S1. The applied voltage and subsequent m/z range depending on the molecular ion of the sample.

Range that contains Molecular Ion of Desired Product within Sample / g mol^{-1}	*Applied Voltage*	*m/z Range*
< 200	10 and 40	50-1200
200 < M < 500	20 and 70	150-1500
500 ≤ M ≤ 1000	40 and 100	200-2000
M ≥1000	40 and 100	200-4000

LCMS

Apparatus: Agilent Technologies 1260 Infinity II. Stationary Phase: Pursuit XRs C18 column (5 µm particle size, 100 Å pore size, 250 x 4.6 mm). Mobile Phase: Solvent A = 0.1% formic acid in water. Solvent B = 0.1% formic acid in acetonitrile. Elution: 0-1 min (5% B), 1-12 min (5-100% B), 12-14 min (100% B), 14-15 min (100%-5% B). Flow rate = 1 ml min^{-1}. UV detection wavelength = 215 (top) and 250 nm (bottom). Mass detection: Single Quadrupole Mass Detector, Model 6125 with nitrogen generator for coupling to PrepLC Agilent System, 100-2000 Da.

MS

All mass spectra not described as high-resolution were obtained by using the conditions shown above for LCMS, with a column bypass so that samples were directly injected into the UV and then mass-detectors.

TLC

TLC was performed on Merck silica gel 60 F_{254} aluminium-backed plates and visualised under a 24 W VL-6.LC UV lamp, using either a 254 or 365 nm wavelength.

Spectroscopic measurements

The absorption of the samples has been measured with a Cary UV-Vis Multicell Peltier (Agilent) spectrometer. The emission was measured by irradiation of the sample in fluorescence quartz cuvettes (width 1 cm) using a NT342B Nd-YAG pumped optical parametric oscillator (Ekspla) at 475 nm.

Luminescence was focused and collected at a right angle to the excitation pathway and directed to a Princeton Instruments Acton SP-2300i monochromator. As a detector, a XPI-Max 4 CCD camera (Princeton Instruments) has been used. Singlet oxygen production was measured with an infrared detector InGaAs (800 - 1550 nm) via the dual network emission monochromator SPEX (600 lines / mm blasé at 1 μm). All spectra were measured using 4-sided quartz cells. $Ru(bpy)_3$ in acetonitrile was chosen as a standard for both fluorescence and singlet oxygen quantum yield determination. Time-resolved experiments were performed using for excitation: a pulsed laser diode emitting at 407 nm (LDH-P-C-400M, FWHM < 70 ps, 1 MHz) coupled with a driver PDL 800-D (both PicoQuant GmbH, BERLIN, Germany) and for detection: an avalanche photodiode SPCM-AQR-15 (EG & G, VAUDREUIL, Canada) coupled with a 650 nm long-wave pass filter as a detection system. The acquisition was performed by a PicoHarp 300 module with a 4-channel router PHR-800 (both PicoQuant GmbH, BERLIN, Germany). The fluorescence decays were recorded using the single-photon counting method. Data were collected up to 1000 counts accumulated in the maximum channel and analyzed using Time Correlated Single Photon Counting (TCSPC) software Fluofit (PicoQuant GmbH, BERLIN, Germany) based on iterative reconvolution using a Levensberg- Marquandt algoritm, enabling the obtention of multi-exponential profiles (mainly one or two exponentials in our cases).

Cell culture

Human ovarian cancer (A2780) cells and mouse fibrosarcoma cells (MCA205) were cultured in RPMI media. Retinal pigment epithelium (RPE-1) cells were cultured in DMEM/F-12 medium. Lung cancer cells (A549) were cultured in F-12K medium. These cell lines were complemented with 10% of fetal bovine serum, 100 U/mL penicillin-streptomycin mixture, and maintained in a humidified atmosphere at 37°C and 5% of CO_2.

(Photo-)cytotoxicity on 2D cell monolayers

The cytotoxicity of the compounds was assessed by measuring cell viability using a resazurin assay. The cells were seeded in triplicates in 96 well plates with a density of 10^4 cells per well in 100 μL of media. After 24 h, the medium was removed, and the cells were treated with increasing concentrations (0.003 → 100 μM) of the compounds diluted in cell media, achieving a total volume of 100 μL/well. The cells were incubated with the compound for 4 h (final incubation time 48 h) or 24 h (final incubation time 72 h). After this time, the media was removed and replaced with 100 μL of fresh medium. For the phototoxicity studies, the cells were exposed to light with an Atlas Photonics LUMOS BIO irradiation system. Each well was constantly irradiated at 670 nm (60 min, 13.50 $J \cdot cm^{-2}$) or 740 nm (60 min, 12.60 $J \cdot cm^{-2}$). During the irradiation, the temperature of the plate was maintained at 37 °C. The cells were grown in the incubator for an additional 44 or 48 h, respectively. For the determination of the dark cytotoxicity, the cells were not irradiated and after the medium exchange directly incubated for 44 h. After this time, the medium was replaced with fresh medium containing resazurin (0.2 mg/mL). After

4 h of incubation, the conversion of resazurin into resorufin was determined upon excitation at 540 nm and measurement of its emission at 590 nm using a Cytation 5 Imaging Reader from BioTek. The obtained data was analysed with the GraphPad Prism software

Generation of 3D MCTS

MCTS were cultured using ultra-low attachment 96 wells plates from Corning (Fisher Scientific 15329740). A2780 cells were seeded at a density of 1,500 cells per well in 100 µL of RPMI. Within 2-3 days, MCTSs have approximately 500 µm of diameter. The formation as well as integrity and diameter of the MCTSs were monitored by microscopy.

(Photo-)cytotoxicity on 3D multicellular tumor spheroids (MCTS)

After 2 days of growing at 37 °C in 5% CO_2, the medium was removed, and MCTSs were treated by adding 100 µL of the medium in the well with increasing concentrations of compounds (**Os-Pt-Cer**, **Os**, Ceritinib, Oxaliplatin, cisplatin, and Ce6 as additional control). For untreated reference MCTSs, only half of the medium was added with fresh medium. After 24 hours, the MCTSs were divided into two identical groups. The first group was strictly kept in the dark. The second group was irradiated at 740 nm for 1 h (12.60 J·cm^{-2}) using a LUMOS-BIO photoreactor (Atlas Photonics). After the irradiation, all groups were incubated additional 7 days. Thereafter, medium was replaced with 100 µL of fresh medium containing resazurin (0.2 mg/mL). After 24 h of incubation at 37 °C, 5% CO_2, plates were read using a SpectraMaxM2 Microplate Reader (λ_{exc} = 540 nm; λ_{read} = 590 nm). Fluorescence data were normalised, and data were fitted using GraphPad Prism Software, and IC_{50} was calculated by non-linear regression. Changes in the size and shape of MCTS were monitored every 24 h using Cytation 5 Imaging Reader from BioTek, objective x4.

Intracellular distribution by confocal fluorescence microscopy

10^4 cells/well (A2780 cells) were seeded in complete medium (RPMI) in µ-slide 8 well (Ibidi) (100 µl/well) and left for 24 h to attach to the bottom. Then, the culture medium was removed, and 100 µl of solution of the corresponding complexes (5 µM) was added. The complexes were incubated with the cells for 4 hours. Thereafter, the medium was removed and replaced by RPMI without phenol red, and Hoechst and MitoTracker Green were added at a final concentration of 1 µg/mL. They were incubated with the cells for 30 min in the dark at 37 ºC. Images were collected in a sequential mode in a Leica TCS SP8 confocal microscope with a 60x oil immersion objective, a line average of 4, and a format of 1024x1024 pixels using excitation wavelength of either 405 nm (Hoechst)→ 420-470 nm, 488 nm (MTG)→ 500-550nm, and 488 nm (complexes)→ 680-730 nm. The confocal pinhole was 1 Airy unit. Images were analysed with LAS X Flim FCS software.

Intracellular distribution by ICP-MS

The localisation of the different parts of the studied complexes was determined by measuring the Os and Pt content inside A2780 cells via ICP-MS. 10^7 cells were incubated with the compound (5 μM) for 4 h at 37°C in the dark. Three independent replicates were made of each of studied conditions. After this time, the cells were trypsinized and counted. In the first portion, the nucleus was extracted using a nucleus extraction kit (Thermo Scientific) using $4\cdot10^6$ cells; in the second portion, the mitochondria were extracted using a mitochondria extraction kit (Thermo Scientific) using 10^7 A2780 cells. In parallel, the content of Os and Pt was determined in a total cell extraction ($2\cdot10^6$ cells). Each sample was digested using a 70% HNO_3 solution overnight at 37ºC. After this time, the solution was removed and washed with ultra-pure water. The Os and Pt content was determined using an ICP-MS apparatus and comparing the results with the Os and Pt calibration standards.

Scratch Assay

A2780 cells were seeded in a 12-well plate to obtain a 90–100% confluency. The cells were incubated for 24 hours to allow them to attach. The cellular monolayer was scratched with a cell scraper, creating a scratch of approximately 1000 μm. Cells were washed twice with PBS, and then 1 mL of the solution containing each studied compound at its IC_{20} value was added to each well. The cells were monitored by imaging over the following time intervals: 0, 6, 24, 32, and 48 h. Agilent BioTek Gen 5 Cytation was used to record the pictures using a 4x objective.

Externalisation of calreticulin

MCA205 and A2780 cells were seeded on μ-slide 8 well (Ibidi) (100 μl/well) and left for 24 h to attach to the bottom at a density of 2 x 10^4 cells/well and incubated overnight. Then, the cells were treated with the tested compounds at their $IC_{25,48h}$ concentrations, and incubated for 16 h. Thereafter, the cells were fixed with formaldehyde (1%), and stained with anti-CRT antibody (ab2907) (Abcam) overnight at 4 °C. Before analysis, samples were co-stained with the secondary antibody for 1 h at room temperature. 30 minutes before starting confocal fluorescence microscopy analysis, Hoechst was added at a final concentration of 1 μg/mL. Images were collected in a sequential mode in a Leica TCS SP8 confocal microscope with a 60x oil immersion objective. Images were analysed with LAS X Film FCS software.

Extracellular ATP release

Extracellular ATP released from treated MCA205 and A2780 cells was detected by the luminescent ATP assay kit (ab113849). Cells were seeded on the 96 well culture plates at a density of 20000 cells/well and incubated overnight. Then, the cells were treated with concentrations of $IC_{25, 48h}$ and $IC_{50, 48h}$ of the tested compounds for 4 h. Thereafter, the medium was replaced, and half of the samples were irradiated at 740 nm for 1 h, meanwhile the others were kept in the dark. After 20 h of incubation, the supernatants from the different samples were transferred to the 96-well black plates (Corning) and

analyzed using an ATP assay kit according to the manufacturer's instructions. Luminescence was measured on a SPARK multimode reader (Tecan). Data were normalized to the untreated control samples.

Detection of extracellular HMGB1 protein

MCA205 and A2780 cells were seeded on μ-slide 8 well (Ibidi) (100 μl/well) and left for 24 h to attach to the bottom at a density of 2 x 10^4 cells/well and incubated overnight. Then, the cells were treated with the tested compounds at their $IC_{25,48h}$ concentrations. 4 hours after the addition, the medium was replaced, and half of the samples were irradiated at 740 nm for one hour, whereas the other half were kept in the dark. After 16 h, the cells were fixed with formaldehyde (1%), and stained with primary antibody (ab79823) overnight at 4 °C. Before analysis, the samples were co-stained with the secondary antibody (ab150079) for 1 h at room temperature. 30 minutes before starting confocal fluorescence microscopy analysis, Hoechst was added at a final concentration of 1 μg/mL. Images were collected in a sequential mode in a Leica TCS SP8 confocal microscope with a 60x oil immersion objective. Images were analysed with LAS X Film FCS software.

Detection of phagocytosis

Phagocytosis was assayed by using the direct co-cultivation of MCA205 and A2780 cells RAW 264.7 with macrophages. Cancer cells were seeded on the 6-well plates (10^5 cells/well) or on μ-slide 8 well (Ibidi, 10^4 cells/well). After 24 hours, cells were treated with the tested compounds at their IC_{25} and $IC_{50,\ 48h}$ concentrations. 4 hours after the addition, the medium was replaced, and half of the samples were irradiated at 740 nm for one hour, whereas the other half were kept in the dark. After 16 h, both cancer and macrophage cell lines were stained with cell trackers. RAW 264.7 macrophages were stained with CellMask Deep Red™ (ThermoFisher Scientific), and MCA205 and A2780 cells were stained with CellTracker Green™ (ThermoFisher Scientific). The cells were co-incubated in a ratio 1:2 (macrophages: cancer cells) for 3 h. After the co-incubation period, samples were harvested and analysed using flow cytometry Attune CytPix (ThermoFisher) and by fluorescence confocal microscopy (Leica TCS SP8) with a ×63/1.40 plan apochromat oil objective. Images were analysed with LAS X Film FCS and ImageJ softwares.

1.1. Procedure and Characterisation

Os1

The synthetic route to this compound is outlined in **Figure S**, where the detailed synthetic conditions can be found in the section below. Compound **1** is literature and was synthesised using conditions previously reported by Mani *et al.* in 2017.[1] The synthesis of **Os** was achieved following a modified version of the synthetic route outlined in the same article. However, in this work, we report the compound with the chloride counterion instead of the hexafluorophosphate counterion.

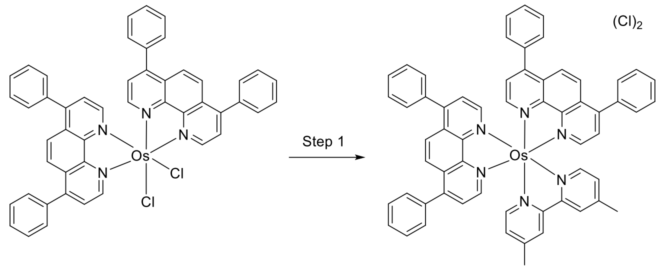

Figure S1. Synthetic route to compound **Os**.

Step 1

A purple suspension of 4,4'-dimethyl-2,2'-dipyridyl (26 mg, 0.143 mmol) and **1** (132 mg, 0.143 mmol) in dry ethylene glycol (20 mL) at 110 °C under pressure for 18 h. To the subsequently cooled brown solution was added saturated NH_4PF_6 (aq), and the isolated precipitate was washed with H_2O (30 mL) and then Et_2O (20 mL, x3) to obtain a dark brown solid. This residue was redissolved in 0.1 % 0.1 M KNO_3 (aq) in acetone (500 mL), slowly loaded onto a pre-equilibrated column flash column cartridge* and eluted with a gradient of 0.1 - 5% 0.1 M KNO_3 (aq) in acetone by automated flash chromatography, to give the desired compound, **Os**, as a nitrate salt with KNO_3 impurities. To the collected product containing fractions was added saturated NH_4PF_6 (aq), and the isolated precipitate was washed with H_2O (30 mL) and then Et_2O (20 mL, x3) to give the desired compound, **Os**, as a pure hexafluorophosphate salt. This solid was re-dissolved in MeOH (2 mL) and to it was added IRA-410 chloride exchange resin (6 g). After stirring this solution at room temperature for 18 h, the supernatant was isolated and reduced *in vacuo*, to give the desired compound, **Os**, with the chloride counterion, as a dark brown solid (153 mg, 97% yield).

* This loading technique is essential to prevent the immediate elution of the osmium complex, i.e. so that it remains at the beginning of the column.

^{1}H NMR: (400 MHz, CD_3CN) δ: 8.54 (s, 2H), 8.23 (d, *J* = 5.5 Hz, 2H), 8.24 – 8.14 (m, 2H), 8.02 (d, *J* = 5.5 Hz, 2H), 7.70 (d, *J* = 5.5 Hz, 2H), 7.68 – 7.54 (m, 22H), 7.49 (d, *J* = 5.5 Hz, 2H), 7.14 (d, *J* = 5.0 Hz, 2H), 2.64 (s, 6H).

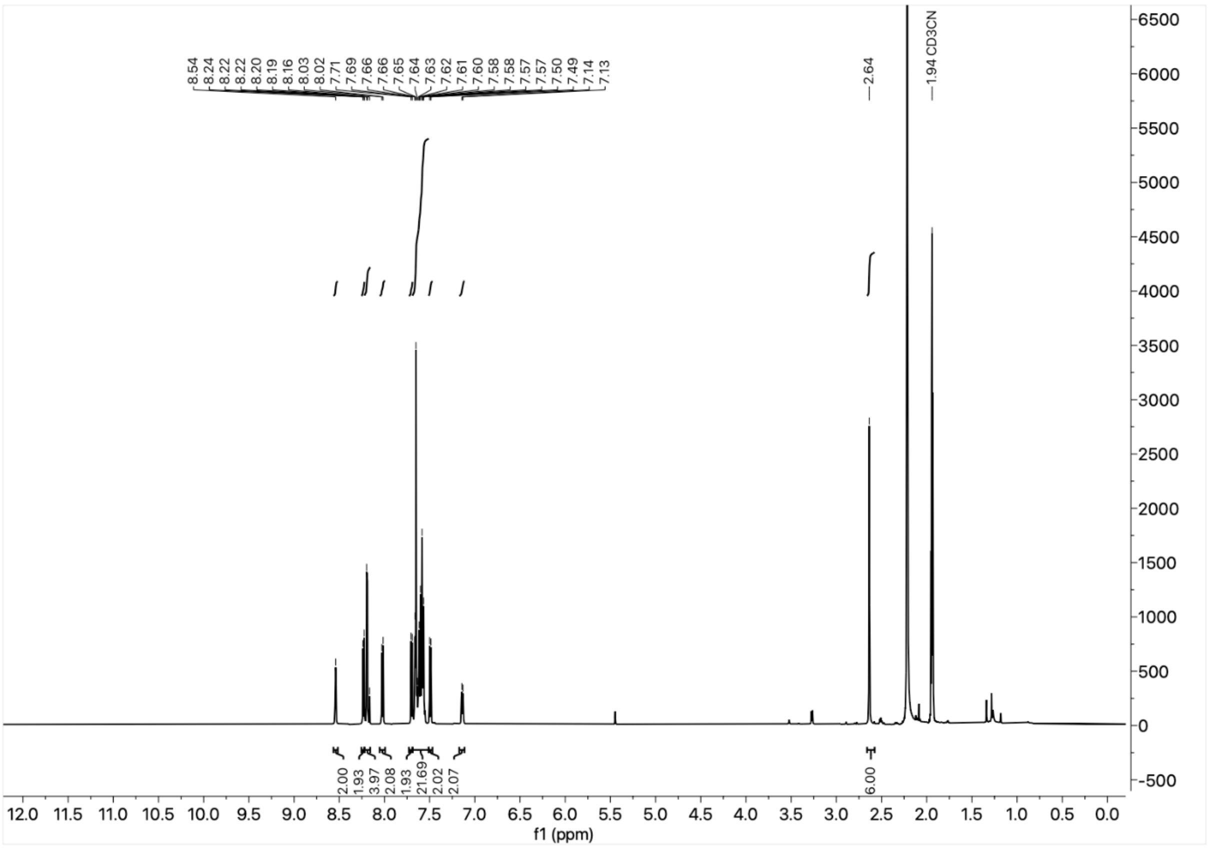

Figure S2. ^{1}H NMR spectrum of **Os** in CD_3CN; water at 2.2 ppm.

^{13}C NMR: (101 MHz, CD_3CN) δ: 159.7, 152.7, 152.4, 151.7, 151.6, 151.4, 151.0, 149.6, 149.5, 136.5, 136.5, 131.1, 131.0, 130.6, 130.6, 130.2, 130.1, 130.1, 129.6, 127.3, 127.2, 126.5, 21.1.

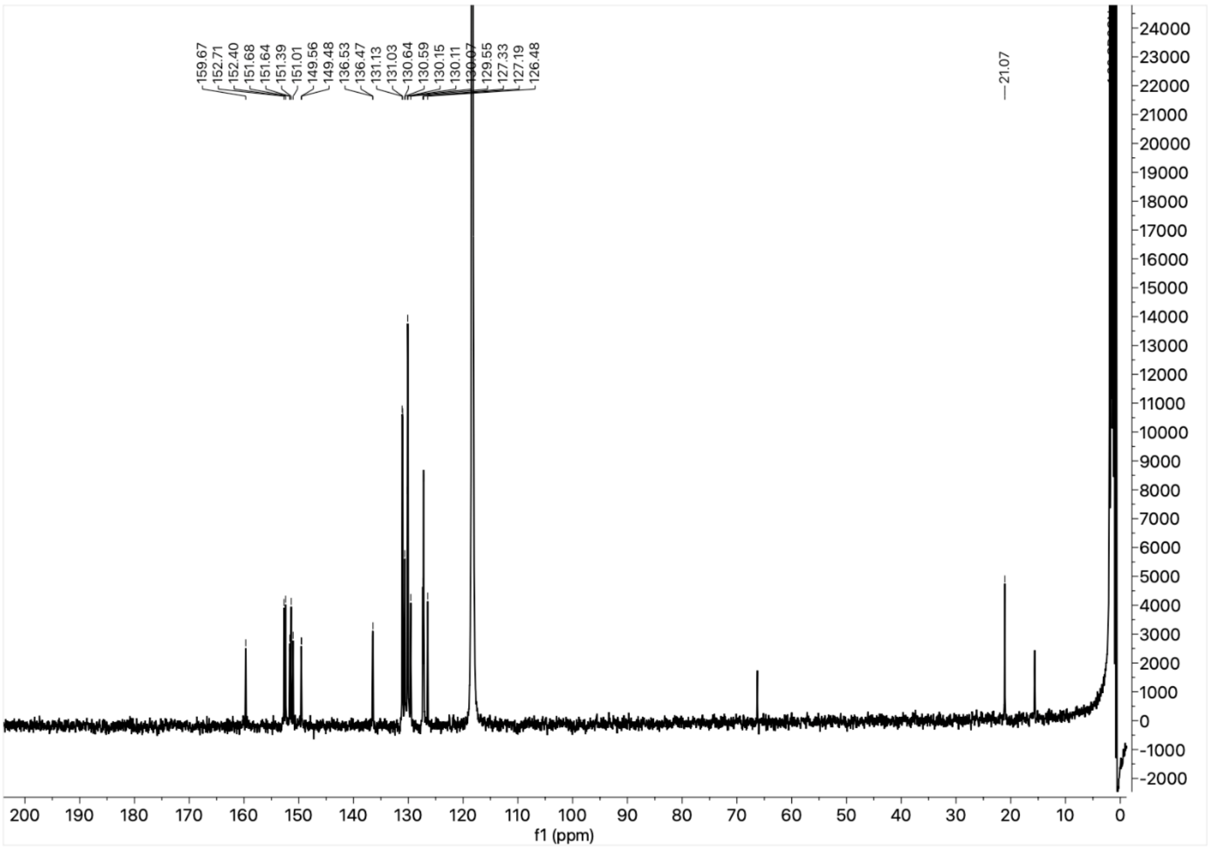

Figure S3. ^{13}C NMR spectrum of **Os** in CD_3CN; diethyl ether at 66 and 16 ppm.

<u>Please Note:</u> ^{19}F and ^{31}P NMR spectra were obtained to confirm the effective removal of the hexafluorophosphate counterion but are omitted for brevity.

LCMS:

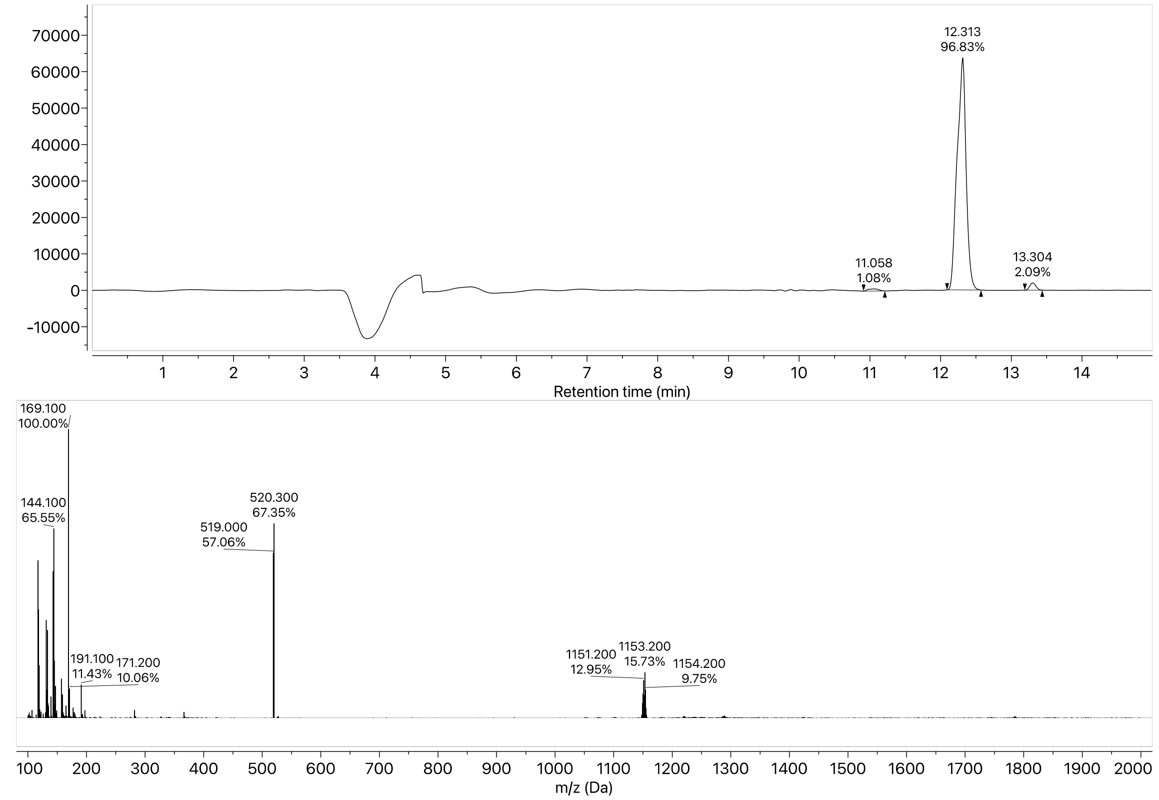

Figure S4. LCMS data of **Os**; with total absorbance chromatogram (top) and corresponding mass spectrum of product peak at 12.3 min (bottom).

Elemental Analysis:

Calculated for [$C_{60}H_{44}Cl_2N_6Os$ • 6 H_2O]: %C 59.16, %H 4.63, %N 6.90
Measured for [$C_{60}H_{44}Cl_2N_6Os$ • 6 H_2O]: %C 58.87, %H 4.06, %N 6.78

<u>Please note:</u> this was accounted for in subsequent assays that required quantitative accuracy.

Os 3

The synthetic route to this compound is outlined in **Figure S**, where the detailed synthetic conditions can be found in the section below.

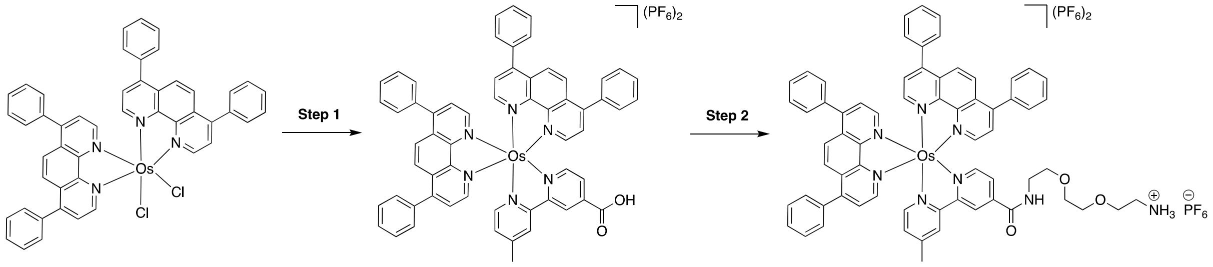

1 2 3

Figure S5. Synthetic route to compound **3**.

Step 1

A purple suspension of 4′-methyl[2,2′-bipyridine]-4-carboxylic acid (36 mg, 0.229 mmol) and **1** (212 mg, 0.229 mmol) in dry ethylene glycol (20 mL) at 110 °C under pressure for 18 h. To the subsequently cooled brown solution was added saturated NH_4PF_6 (aq), and the isolated precipitate was washed with H_2O (30 mL). This residue was redissolved in dichloromethane (200 mL), slowly loaded onto a pre-equilibrated flash column cartridge* and eluted with a gradient of 0 - 7% MeOH in dichloromethane by automated flash chromatography. To the collected product containing fractions was added saturated NH_4PF_6 (aq), and the isolated precipitate was washed with H_2O (30 mL) and then Et_2O (20 mL, x3) to give the desired compound, **2,** as a crude mixture.

* This loading technique is essential to prevent the immediate elution of the osmium complex. I.e. So that it remains at the beginning of the column.

LCMS:

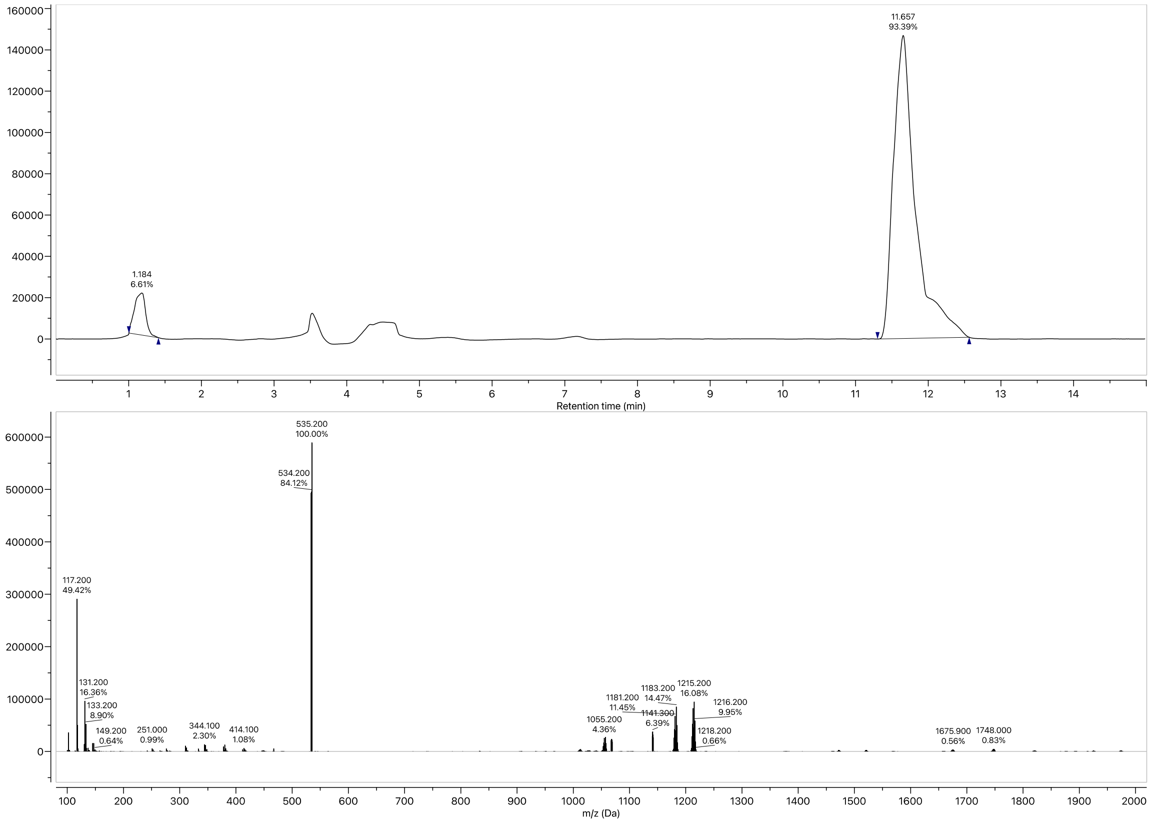

Figure S6. LCMS data of **2**; with total absorbance chromatogram (top) and corresponding mass spectrum of product peak at 11.657 min (bottom). Unidentified impurity appears as a shoulder on the right-hand side of the product peak.

Step 2

A dark brown solution of **2** (65 mg, 0.0478 mmol), *tert*-Butyl [2-[2-(2-aminoethoxy)ethoxy]ethyl]carbamate (12 mg, 0.0478 mmol), *N*-(3-Dimethylaminopropyl)-*N'*-ethylcarbodiimide hydrochloride (14 mg, 0.717 mmol), 1-hydroxybenzotriazole hydrate (11 mg, 0.0717 mmol), DIPEA (18 µL, 0.0956 mmol) was stirred at room temperature in dichloromethane (20 mL) for 18 h. TLC analysis confirmed complete consumption of both starting materials. To the reaction mixture was added trifluoroacetic acid (3 mL). After stirring at room temperature for 4 h, the mixture was dried *in vacuo* by co-evaporation with dichloromethane (10 mL, x3). The resulting residue was re-dissolved in MeCN (2mL), precipitated with saturated NH_4PF_6 (aq), and the isolated precipitate washed with H_2O (30 mL) and then Et_2O (20 mL, x3) to give the desired compound, **3,** as a dark brown solid (60 mg, 16% yield over two steps).

^{1}H NMR: (400 MHz, CD_2Cl_2) δ: 8.79 (d, *J* = 2.0 Hz, 1H), 8.56 (s, 1H), 8.28 – 8.18 (m, 5H), 8.16 (d, *J* = 5.5 Hz, 1H), 7.96 (dd, *J* = 5.5, 0.5 Hz, 2H), 7.86 (d, *J* = 6.0 Hz, 1H), 7.77 (d, *J* = 5.5 Hz, 1H), 7.73 (d, *J* = 5.5 Hz, 1H), 7.69 – 7.47 (m, 26H), 7.15 (dd, *J* = 6.0, 2.0 Hz, 1H), 5.31 – 5.30 (m, 2H), 3.77 – 3.52 (m, 12H), 3.24 (dd, *J* = 6.0, 4.0 Hz, 2H), 2.68 (s, 3H).

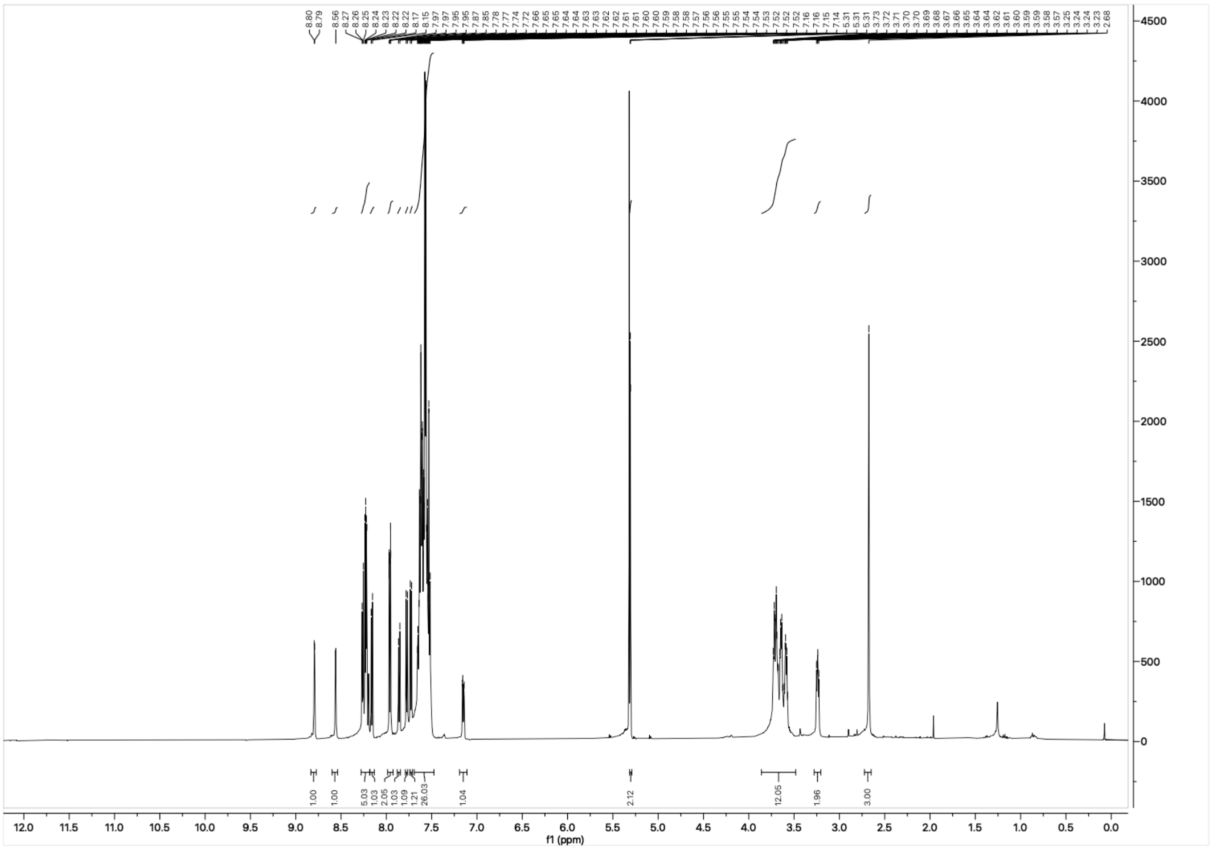

Figure S7. ^{1}H NMR spectrum of **3** in CD_2Cl_2.

^{13}C NMR: (101 MHz, CD_2Cl_2) δ: 164.7, 160.1, 158.3, 151.7, 151.4, 151.3, 151.1, 150.8, 150.5, 150.5, 150.1, 150.1, 149.8, 149.6, 149.4, 149.3, 149.2, 141.2, 135.3, 135.2, 135.1, 130.2, 130.1, 129.9, 129.8, 129.4, 129.3, 129.2, 127.2, 126.9, 126.6, 126.5, 126.4, 126.1, 121.6, 70.4, 70.2, 69.8, 66.1, 40.5, 40.4, 20.7.

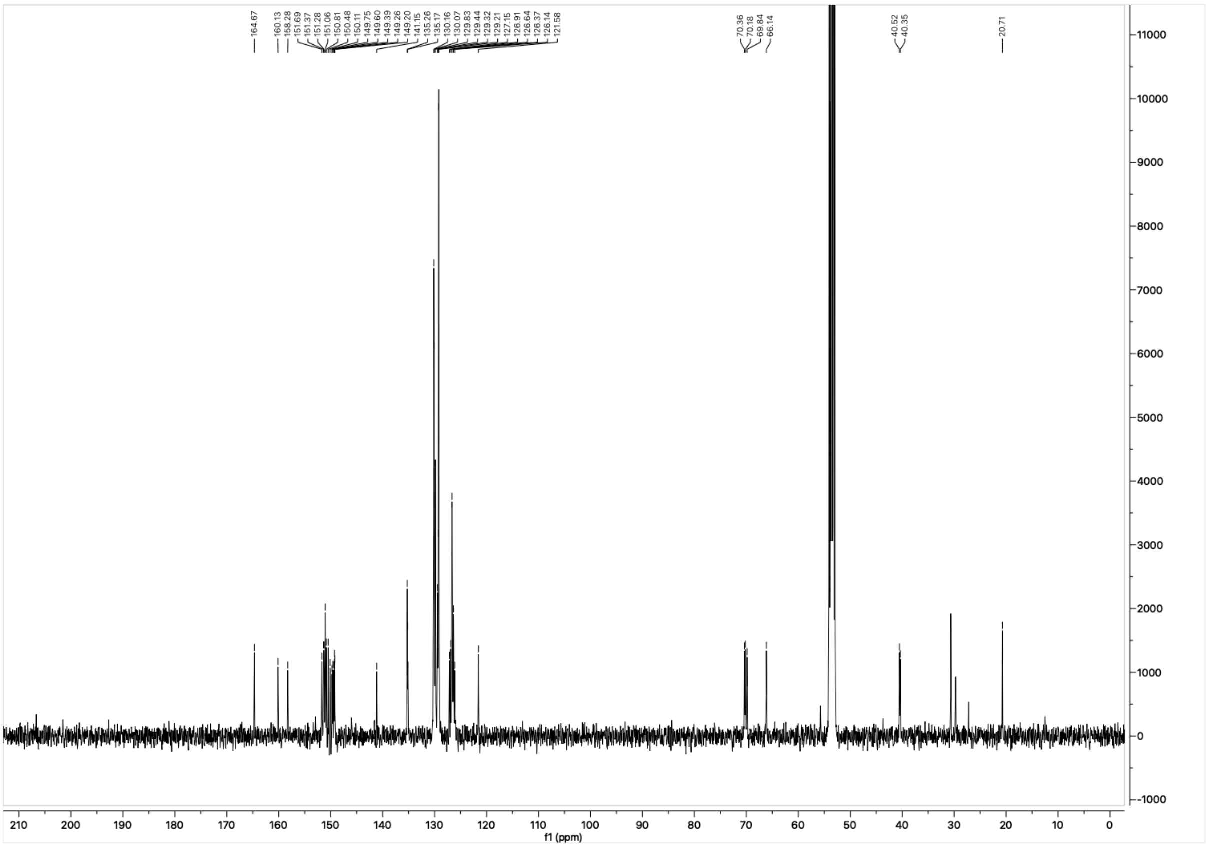

Figure S8. ^{13}C NMR spectrum of **3** in CD_2Cl_2.

LCMS:

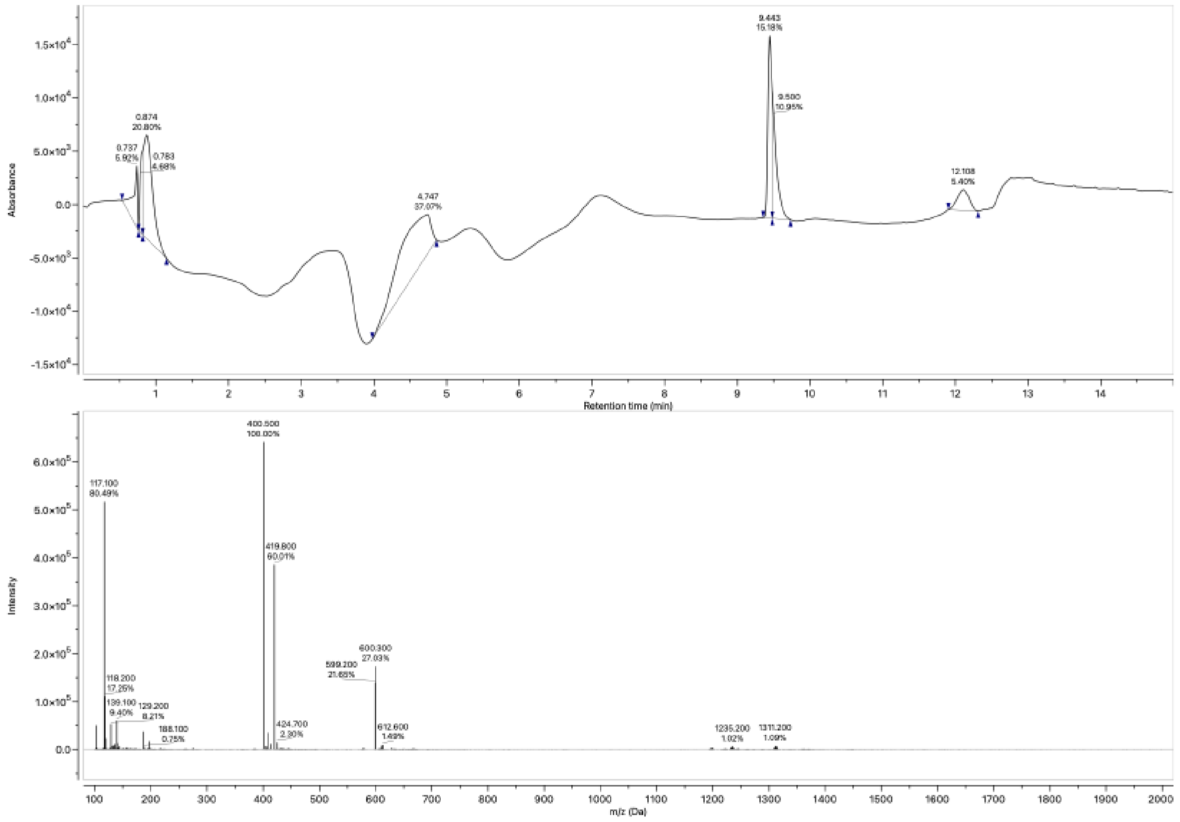

Figure S9. LCMS data of **3**; with total absorbance chromatogram (top) and corresponding mass spectrum of product peak at 9.443 min (bottom).

Os-Pt and Pt-Ceritinib

The synthetic scheme of **Os-Pt** and **Pt-Ceritinib**.

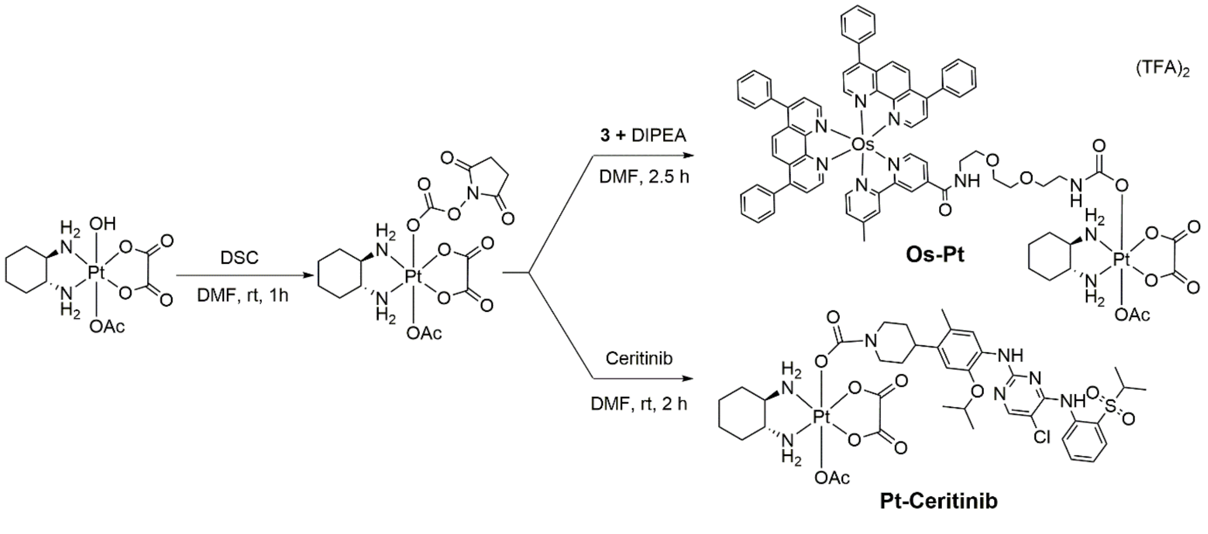

Figure S10: Synthetic route of **Os-Pt** and **Pt-Ceritinib**.

Synthesis of *ctc*-[Pt(DACH)(MSC)(OAc)(Ox)]

ctc-[Pt(DACH)$_2$(OAc)(OH)(Ox)] (165 mg, 0.35 mmol) was stirred with 125 mg of disuccinimidyl carbonate, DSC (0.49 mmol, 1.4 equiv) in 2mL of DMF for 1h at room temperature. The DMF was evaporated in reduced pressure and the sticky residue was re-suspended in acetonitrile and precipitated with diethyl ether. The precipitate was collected by centrifugation and washed twice with diethyl ether and used for the next step without further purification. Yield: 195 mg (91%). This activated Pt(IV) precursor *ctc*-[Pt(DACH)(MSC)(OAc)(Ox)] was used in next steps to synthesize both **Os-Pt** and **Pt-Ceritinib** complexes.

Os-Pt: To the solution of complex **3** (22 mg, 0.0134 mmol) in DMF (1 mL), di-isopropylethyl amine (DIPEA) was added and stirred at room temperature for 15 min. [Pt(DACH)(OAc)(MSC)(Ox)] (16.5 mg, 0.027 mmol) in 1 mL DMF was added to this and stirred at room temperature. The progress of the reaction was monitored by HPLC. After 2 h the reaction mixture was concentrated and diluted with methanol. The final product was isolated by injecting the solution into preparative HPLC using 0.1% TFA in water and acetonitrile as a gradient. The product was concentrated and lyophilized. Yield: 18.2 mg (67%). RT = 5.49 min (Purity: 97%); ESI-HRMS (+ve mode): m/z calculated for $[C_{77}H_{72}N_{10}O_{11}OsPt]^{2+}$ 849.7316 found : 849.7304.

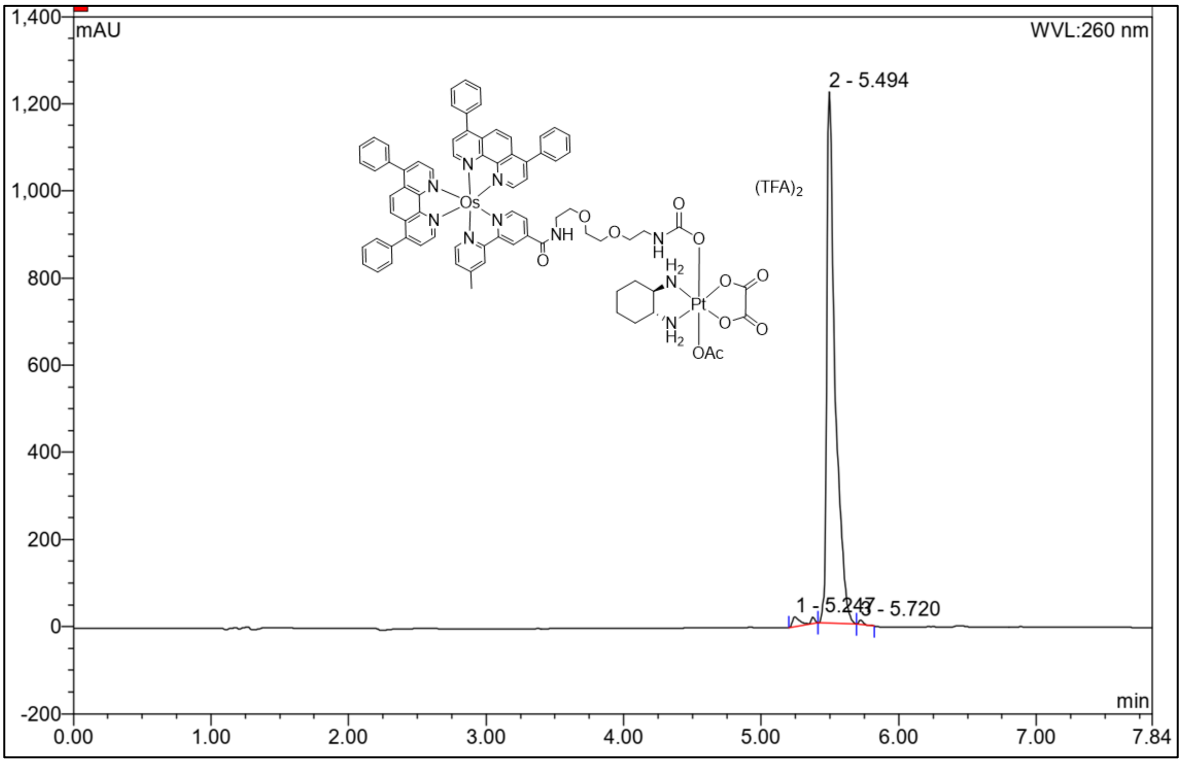

Figure S11. HPLC chromatogram of **Os-Pt** ran with 0 – 100% linear gradient of acetonitrile in 0.1% TFA in water over 5.84 min + 2 min constant 100% acetonitrile.

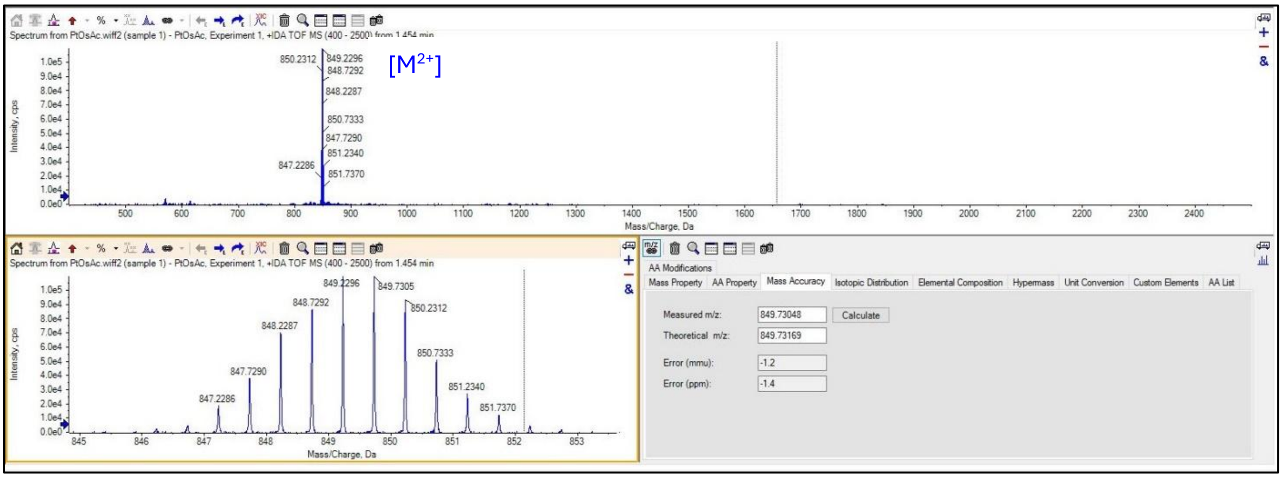

Figure S12. ESI-HRMS (+ve mode) of **Os-Pt**.

Pt-Ceritinib: To the solution of Ceritinib (42 mg, 0.075 mmol) in DMF (1.5 mL), activated [Pt(DACH)(OAc)(MSC)(Ox)] (50 mg, 0.081 mmol) was added after dissolving in 1 mL DMF and stirred at room temperature. The progress of the reaction was monitored by HPLC. After 2 h the reaction mixture was concentrated and diluted with methanol (1:3 DMF/Methanol). The final product was isolated by injecting the solution into preparative HPLC using 0.1% TFA in water and acetonitrile as a gradient. The product was concentrated and lyophilized. Yield: 48.1 mg (60%). RP-HPLC (Analytical): RT = 5.42 min (Purity: >99%); ESI-MS (+ve mode): m/z calculated for $[C_{39}H_{53}ClN_7O_{11}PtS]^+$ 1057.28 found: 1058.53.

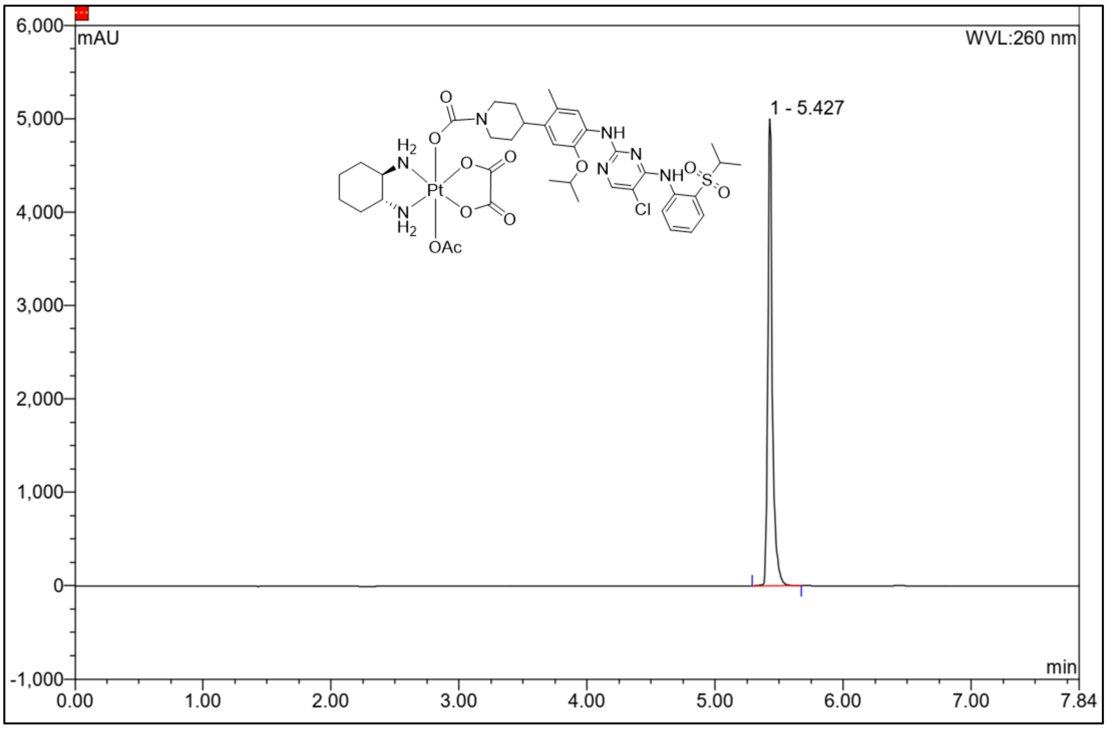

Figure S13. HPLC chromatogram of complex **Pt-Ceritinib** ran with 0 – 100% linear gradient of acetonitrile in 0.1% TFA in water over 5.84 min + 2 min constant 100% acetonitrile.

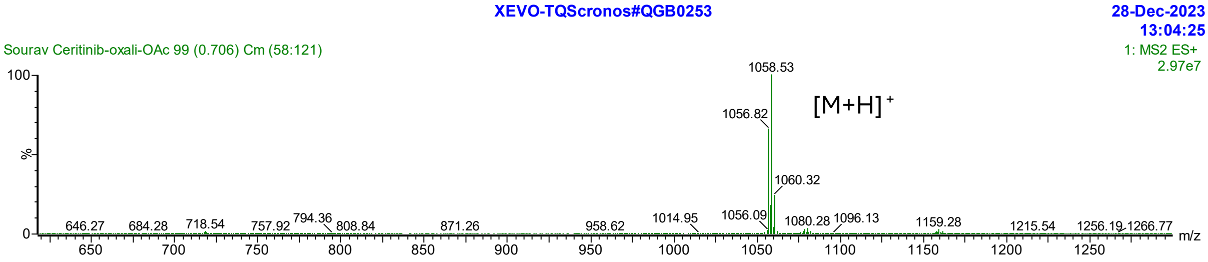

Figure S14. ESI-MS (+ve mode) of **Pt-Ceritinib**.

Os-Pt-Cer

The synthetic scheme of **Os-Pt-Ceritinib** is outlined in **Scheme 1** with detailed synthetic conditions. Oxaliplatin diOH (140 mg, 0.324 mmol) was stirred overnight with 83 mg of disuccinimidyl carbonate, DSC (0.324 mmol) in 15 mL of DMSO at room temperature. Upon completion of the reaction, as indicated by ^{195}Pt NMR, the reaction mixture was filtered, and the filtrate was treated with large amounts of diethyl ether, affording a two-phase system. The ether phase was removed after centrifugation. The procedure was repeated several times until a sticky yellow solid was obtained. It was then suspended in a minimum amount of acetonitrile and then precipitated in diethyl ether. The precipitate was collected by centrifugation and washed with diethyl ether and used for the next step without further purification. Yield: 124 mg (66.7%). To the solution of Ceritinib (55.8 mg, 0.1 mmol) in DMF (2 mL) activated *ctc*-[Pt(DACH)(MSC)(OH)(Ox)] (63 mg, 0.11 mmol) in 2 mL DMF was added and stirred at room temperature for 4 h. The formation of a new peak at RT = 5.1 min was observed by analytical HPLC. The reaction mixture was concentrated by evaporation and diluted with acetonitrile before being injected into preparative HPLC. The product was isolated by injecting this solution into preparative HPLC using 0.1% TFA in water and acetonitrile as a gradient. The product isolated was concentrated and lyophilised, and used for the next step. Yield: 65 mg (64%).

ctc-[Pt(DACH)(Ceritinib)(OH)(Ox)] (50 mg, 0.049 mmol) was then reacted with 19 mg of DSC (0.074 mmol, 1.5 equiv) in 1.5 mL of DMF for 1h at room temperature. The DMF was evaporated in reduced pressure, and the sticky residue was re-suspended in acetonitrile and precipitated in diethyl ether. The precipitate of *ctc*-[Pt(DACH)(Ceritinib)(MSC)(Ox)] was collected by centrifugation and washed with diethyl ether and used for the next step without further purification. Yield: 48 mg (84.3%). To the solution of complex **3** (50 mg, 0.0305 mmol) in DMF (2 mL), DIPEA (0.032 mmol, 5.6 µL, 1.05 eq.) was added and stirred at room temperature for 15 min. *ctc*-[Pt(DACH)(Ceritinib)(MSC)(Ox)] (47.7 mg, 0.0412 mmol, 1.35 eq.) was then dissolved in 1 mL DMF and added to this solution and stirred at room temperature. The progress of the reaction was monitored by HPLC. The new peak appeared at RT = 6.22 min (analytical HPLC). After 2h the reaction mixture was concentrated and diluted with methanol. The final product was isolated by injecting the solution into preparative HPLC using 0.075% TFA in water and acetonitrile as a gradient. The product was concentrated and lyophilized. Yield: 61 mg

(80.8%). RT = 6.22 min (Purity: >99%); ESI-HRMS (+ve mode): m/z calculated for $[C_{104}H_{104}ClN_{15}O_{14}OsPtS]^{2+}$ 1120.8296 found : 1120.8274 and for $[C_{104}H_{105}ClN_{15}O_{14}OsPtS]^{3+}$ 747.5556 found 747.5540.

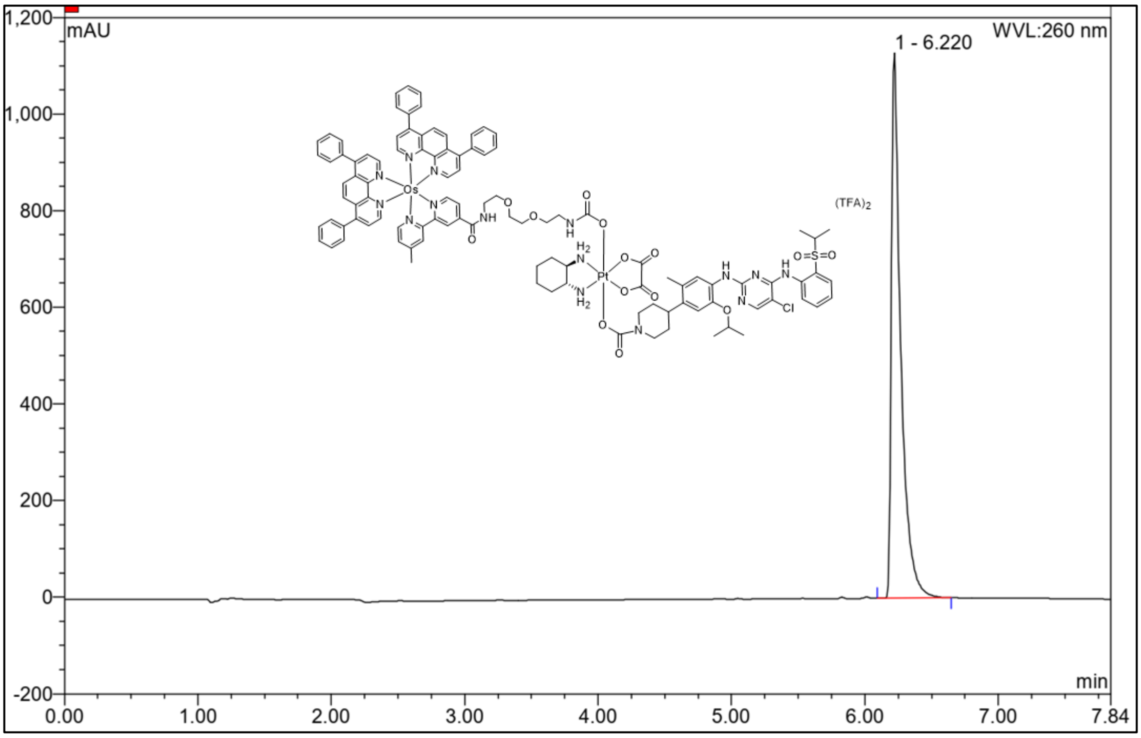

Figure S15. HPLC chromatogram of **Os-Pt-Ceritinib** ran with 0 – 100% linear gradient of acetonitrile in 0.1% TFA in water over 5.84 min + 2 min constant 100% acetonitrile.

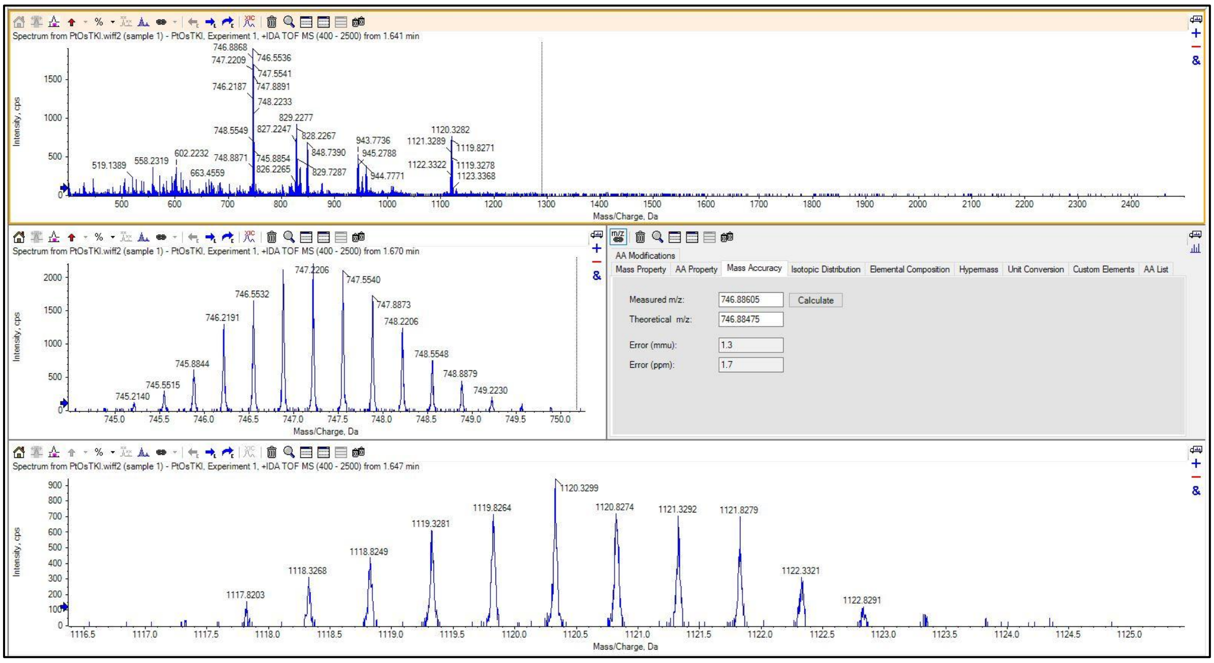

Figure S16. ESI-HRMS (+ve mode) of complex **Os-Pt-Ceritinib**.

Spectroscopic measurements

Table S2. Photophysical data of the studied compounds were measured in an aerated CH_3CN solution. The lifetimes were measured at room temperature, with a concentration of oxygen in the samples of around 21% of the saturation. The osmium complex (**Os**) presents a bi-exponential decay, whereas the other complexes (**Os-Pt** and **Os-Pt-Cer**) present mono-exponential decays. The lifetimes are around 40 ns for the osmium complexes and 160 ns for tris(2,2'-bipyridyl) ruthenium (II).

	UV/vis ×10^3 (ε [dm^3 mol^{-1} cm^{-1}])	*λ_{em} (λ_{ex}) [nm]*	*Lifetimes [ns] / (χ^2)*
Os-Pt-Cer	290 (26.2); 445 (6.3); 500 (5.4); 590 (1.6)	740 (475)	39.6 / 0.999
Os-Pt	290 (9.5); 445 (4.7); 500 (4.2); 590 (0.1)	745 (475)	39.4 / 0.950
Os	290 (44.0); 445 (15.7); 500 (14.1); 590 (4.9)	730 (475)	42 (27%) - 9,3 (73%) / 0.839
Pt-Cer	305 (10.5)	-	
Pt(OAc)₂	285 (10.0)	-	
Ceritinib	305 (4.0)	-	

Table S3. Singlet oxygen production. Quantum yield and singlet oxygen quantum yield after excitation of the studied osmium complexes after excitation at 450 nm in acetonitrile. Tris(2,2'-bipyridyl) ruthenium (II) was used as a standard.

	Quantum yield	*Singlet oxygen quantum yield*
Os-Pt-Cer	< 0.01	0.11
Os-Pt	< 0.01	0.11
Os	< 0.01	0.13
$Ru(bpy)_3$	0.077	0.77

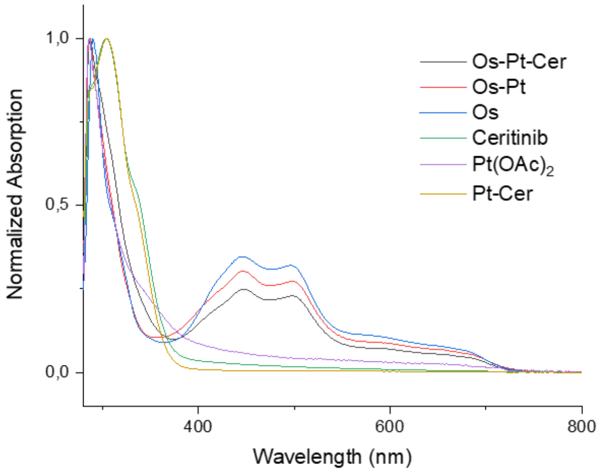

T

Figure S17. Normalized absorption spectrum of the studied compounds in acetonitrile.

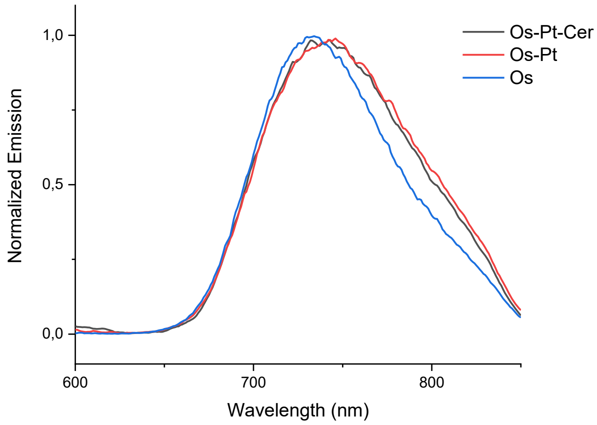

Figure S18. Normalized emission spectrum of **Os**, **Os-Pt**, and **Os-Pt-Cer** in acetonitrile. Excitation wavelength: 475 nm.

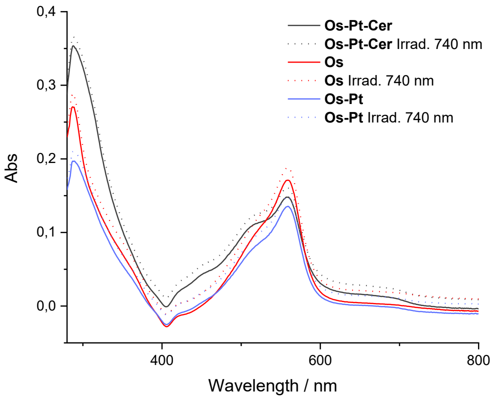

Figure S19. Absorption spectra of **Os**, **Os-Pt**, and **Os-Pt-Cer** complexes in complete RPMI medium, before and after irradiation at 740 nm (12.60 J/cm^2, 1 h).

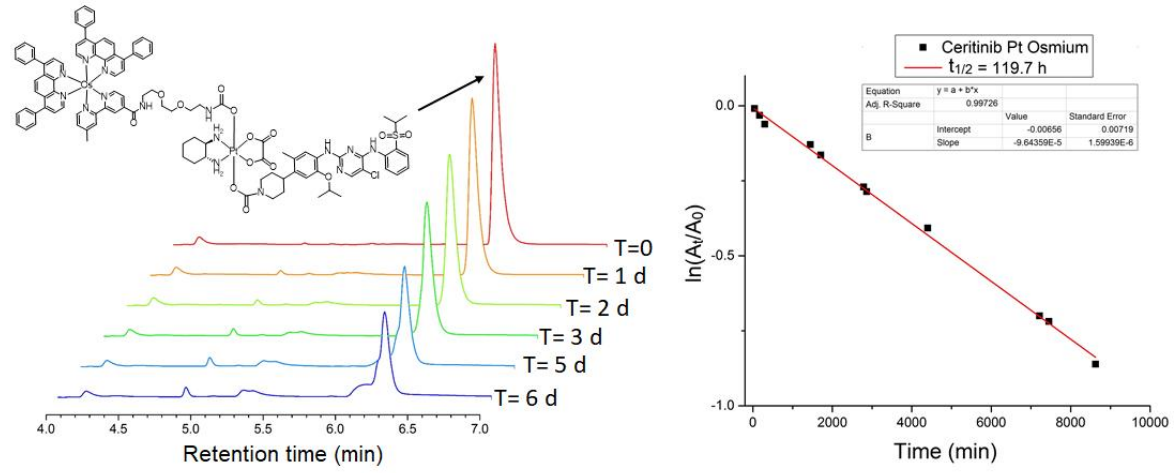

Figure S20. Stability of **Os-Pt-Ceritinib** in dark in 1:9 (v/v) DMSO and RPMI with serum, monitored by HPLC chromatogram for 6 days at 37°C. $t_{1/2}$ (stability) = 5 days.

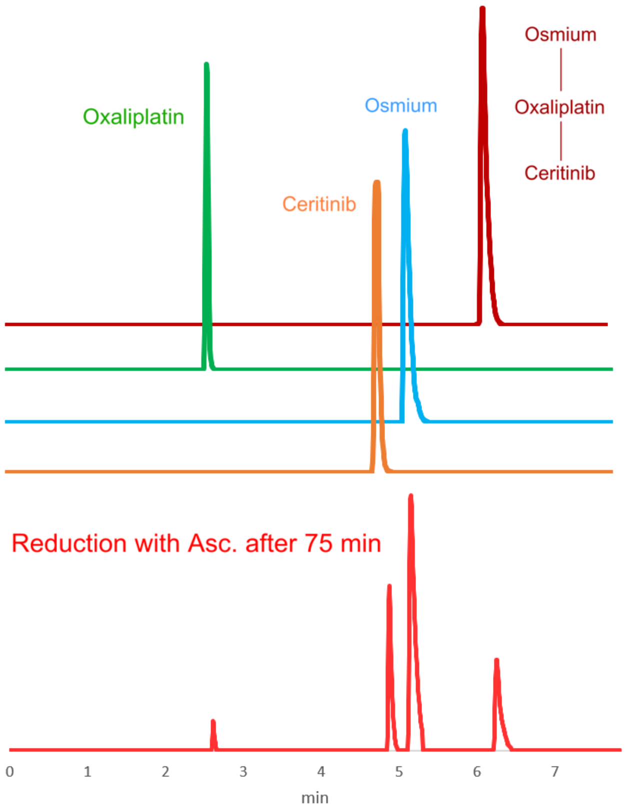

Figure S21. Proof of concept: release of free Ceritinib (**L**) and Os-amine (**3**) from the **Os-Pt-Ceritinib** prodrug (**6**) after reduction with 10 eq. ascorbic acid.

Table S4. IC_{50} (μM) values in normoxic conditions, in the dark and upon irradiation at 670 nm (60 min, 13.50 J/cm2) on A2780 cells after 48 h of incubation. Average of three independent measurements.

	A2780		
**Compound**	**Dark**	**Irrad. 670 nm**	**PI**
Os-Pt-Ceritinib	9.40 ± 0.06	0.28 ± 0.02	34
Os-Pt	11.50 ± 0.1	0.24 ± 0.04	48
Os	39.24 ± 0.2	0.53 ± 0.02	74
Pt-Ceritinib	>100	-	-
Pt (OAc)$_2$	41.9 ± 0.9	-	-
Ceritinib	2.61 ± 0.5	-	-
Oxaliplatin	46.15 ± 0.2	-	-
PPIX	> 100	1.93 ± 0.05	-
Cisplatin	1.92 ± 0.4	-	-

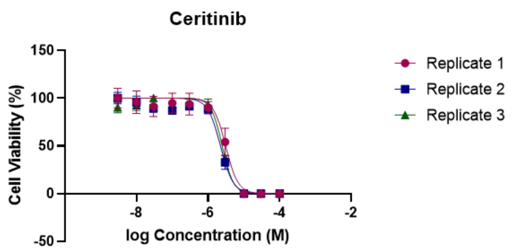

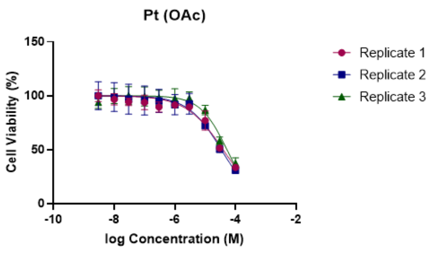

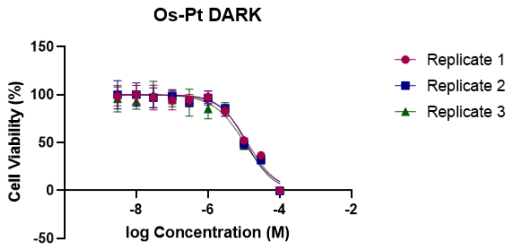

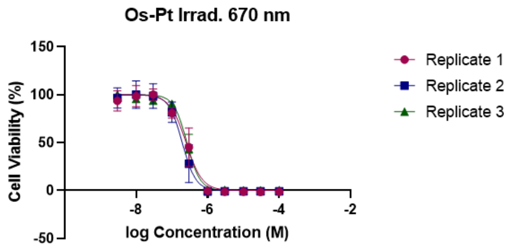

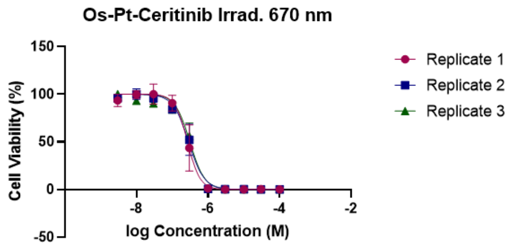

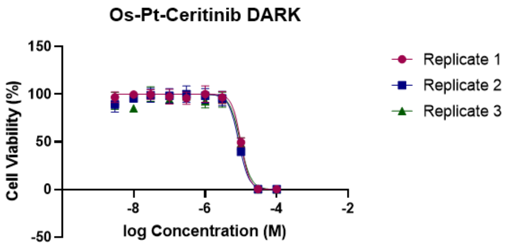

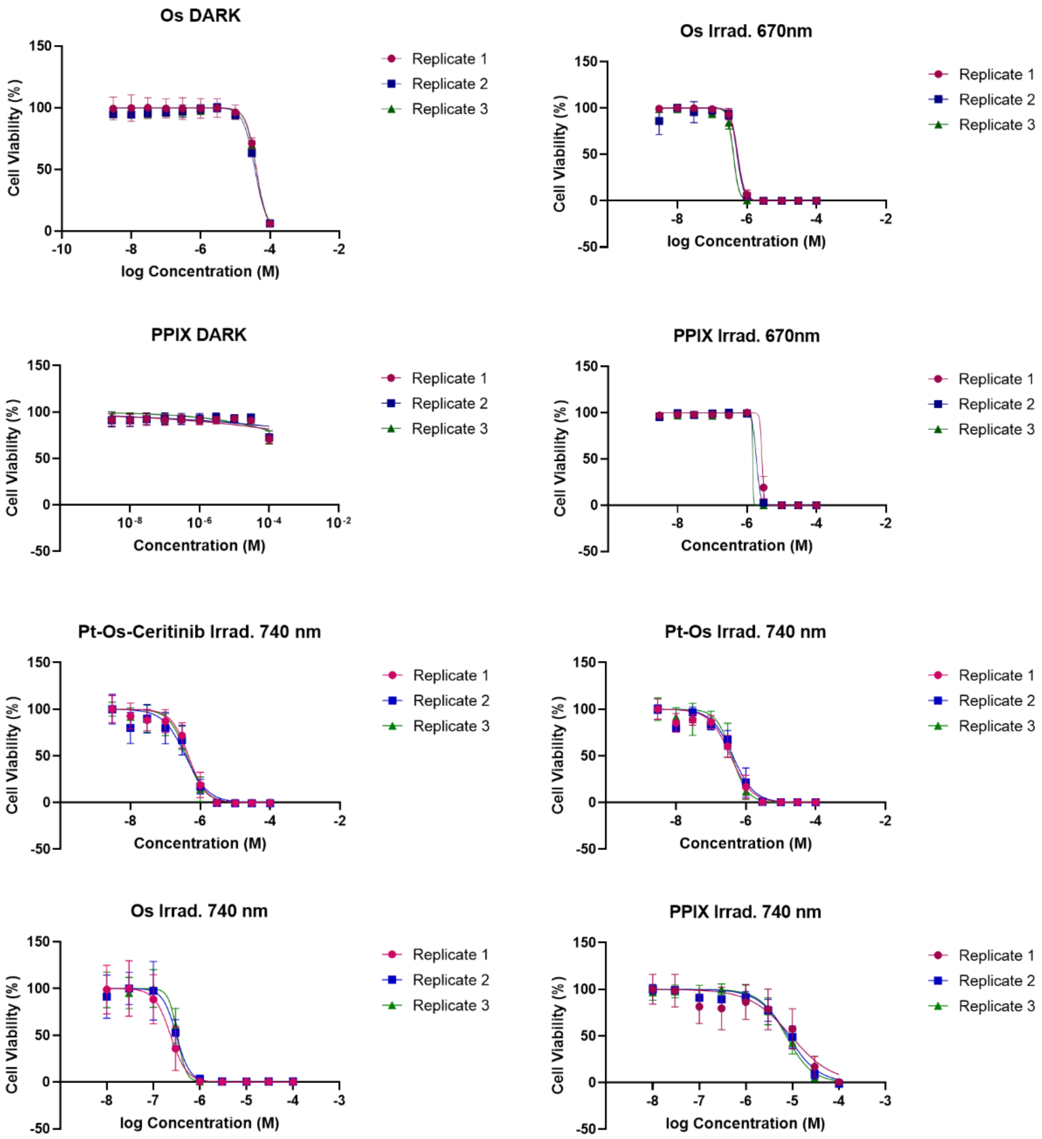

Figure S22. IC_{50} curves of studied compounds were obtained after resazurin cell viability assay in A2780 cell line at 48 h, in the dark, and irradiated at 670 and 740 nm.

Pt-Os-Ceritinib RPE-1 Irrad. 740 nm

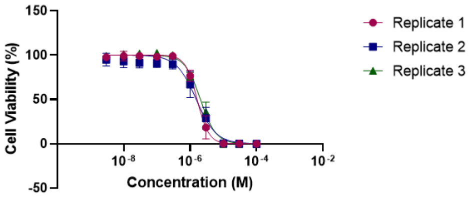

Pt-Os-Ceritinib RPE-1 Dark

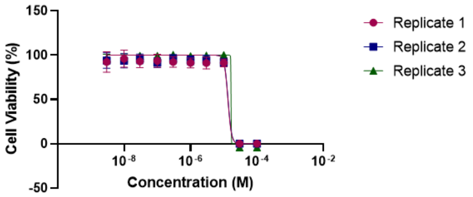

Pt-Os RPE-1 Irrad. 740 nm

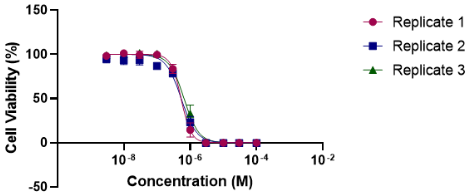

Pt-Os RPE-1 Dark

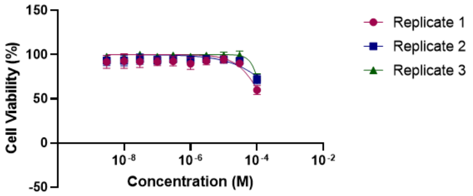

PPIX RPE-1 DARK

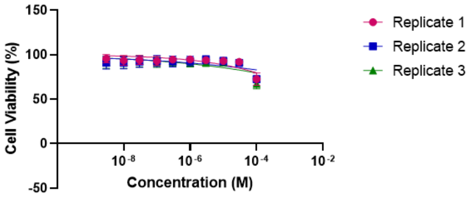

PPIX RPE-1 Irrad. 740 nm

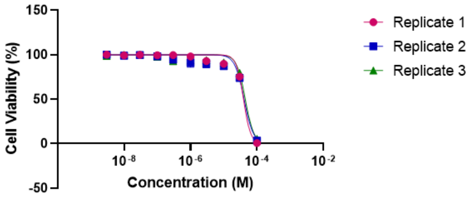

Os RPE-1 DARK

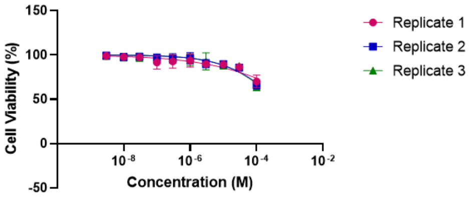

Os RPE-1 Irrad. 740 nm

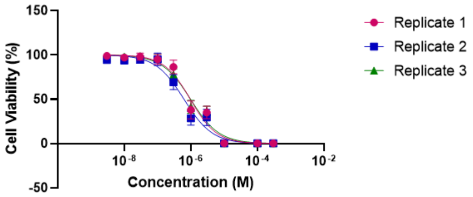

Pt-Ceritinib RPE-1

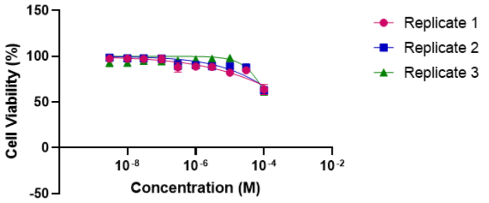

PPIX RPE-1 Irrad. 740 nm

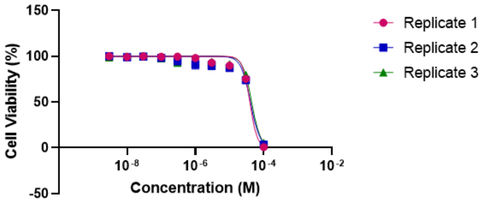

Pt(OAc) RPE-1

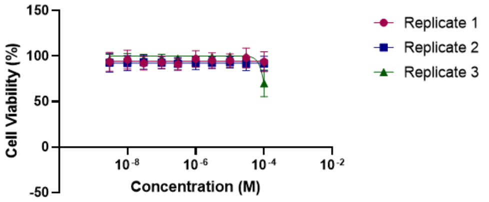

Os RPE-1 Irrad. 740 nm

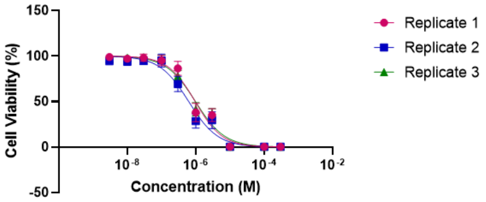

Oxaliplatin RPE-1

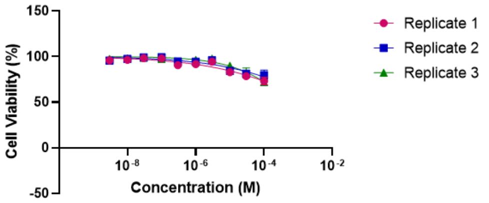

Ceritinib RPE-1

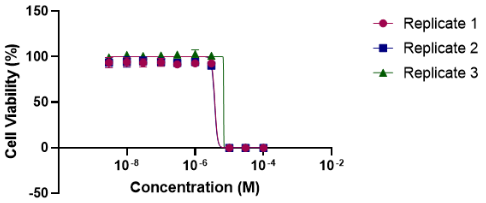

Figure S23. IC_{50} curves of studied compounds were obtained after resazurin cell viability assay in RPE-1 cell line at 48 h, in the dark, and irradiated at 740 nm.

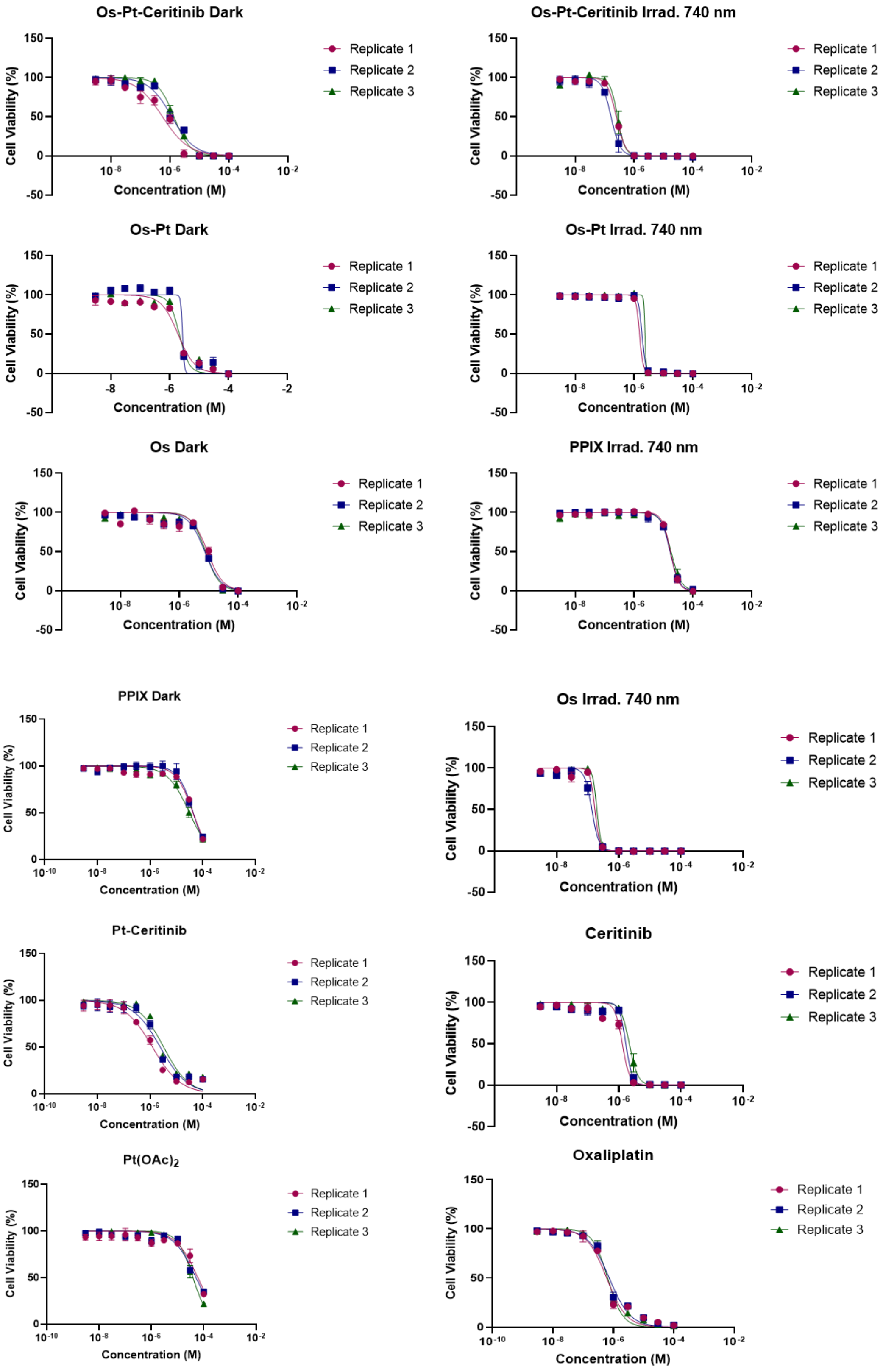

Figure S24. IC_{50} curves of studied compounds were obtained after resazurin cell viability assay in A2780 cell line at 72 h, in the dark, and irradiated at 740 nm.

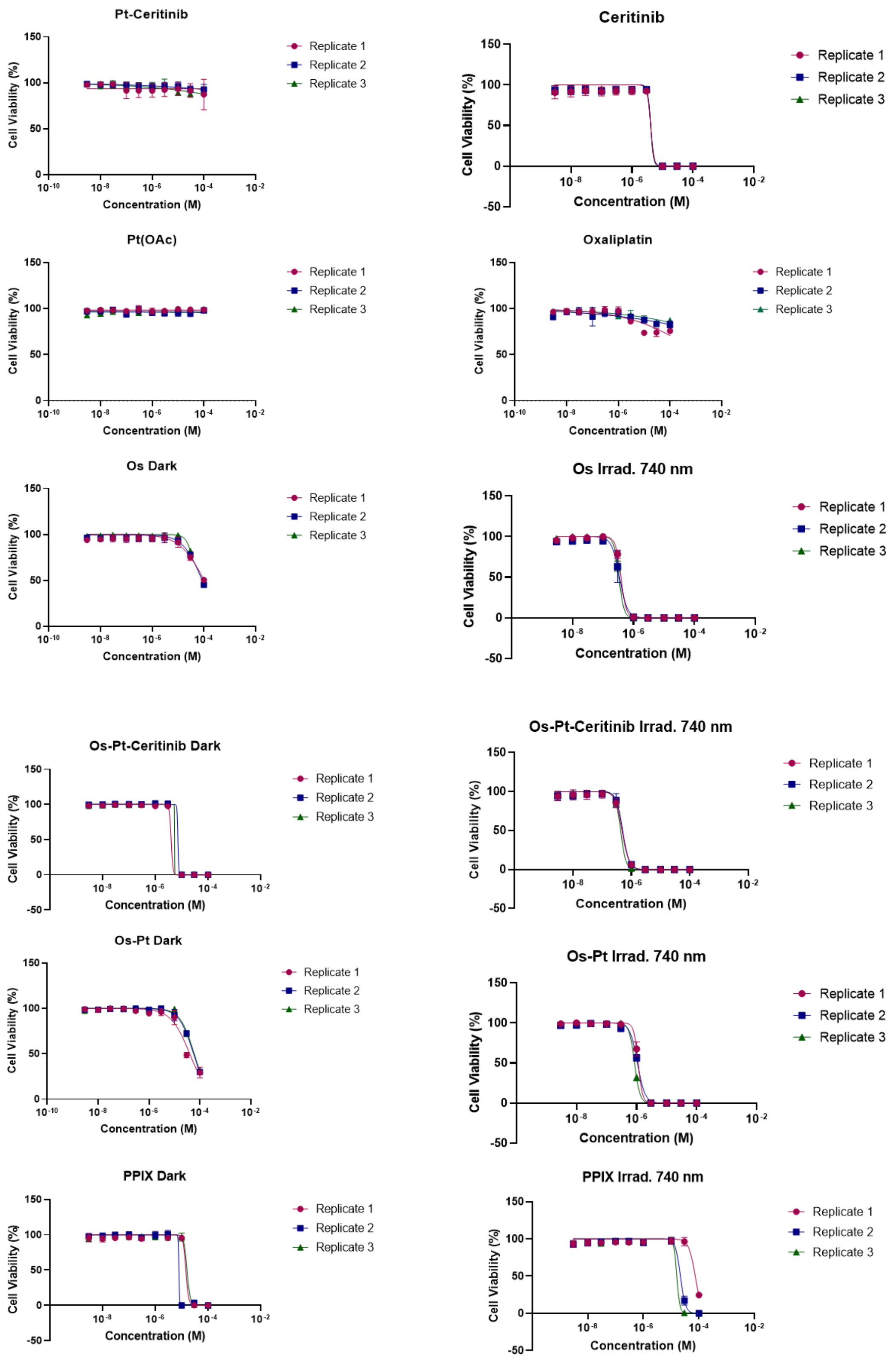

Figure S25. IC_{50} curves of the studied compounds were obtained after resazurin cell viability assay in RPE-1 cell line at 72 h, in the dark, and irradiated at 740 nm.

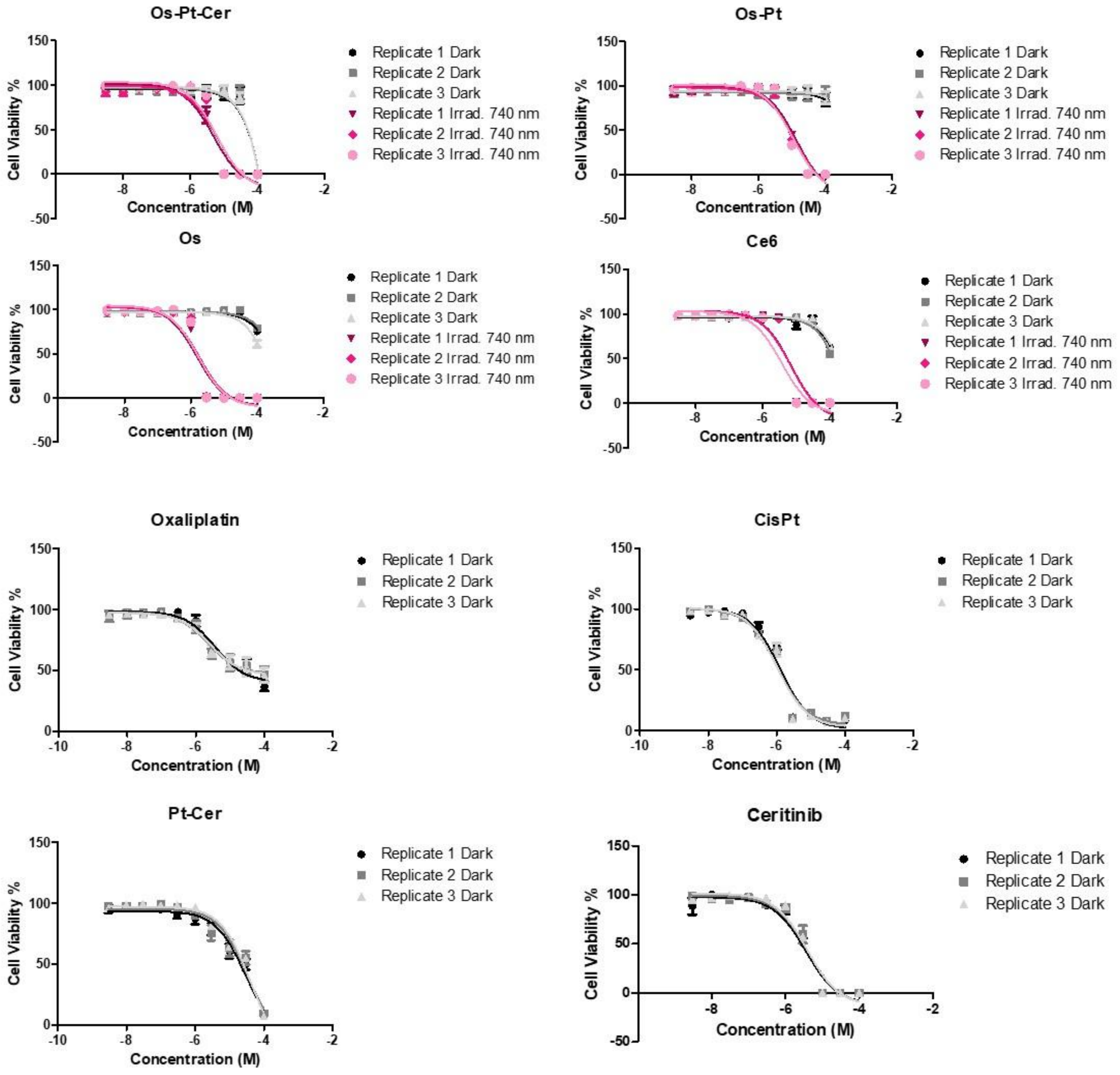

Figure S26. IC_{50} curves of studied compounds were obtained after resazurin cell viability assay in A549 cell line at 48 h, in the dark, and irradiated at 740 nm.

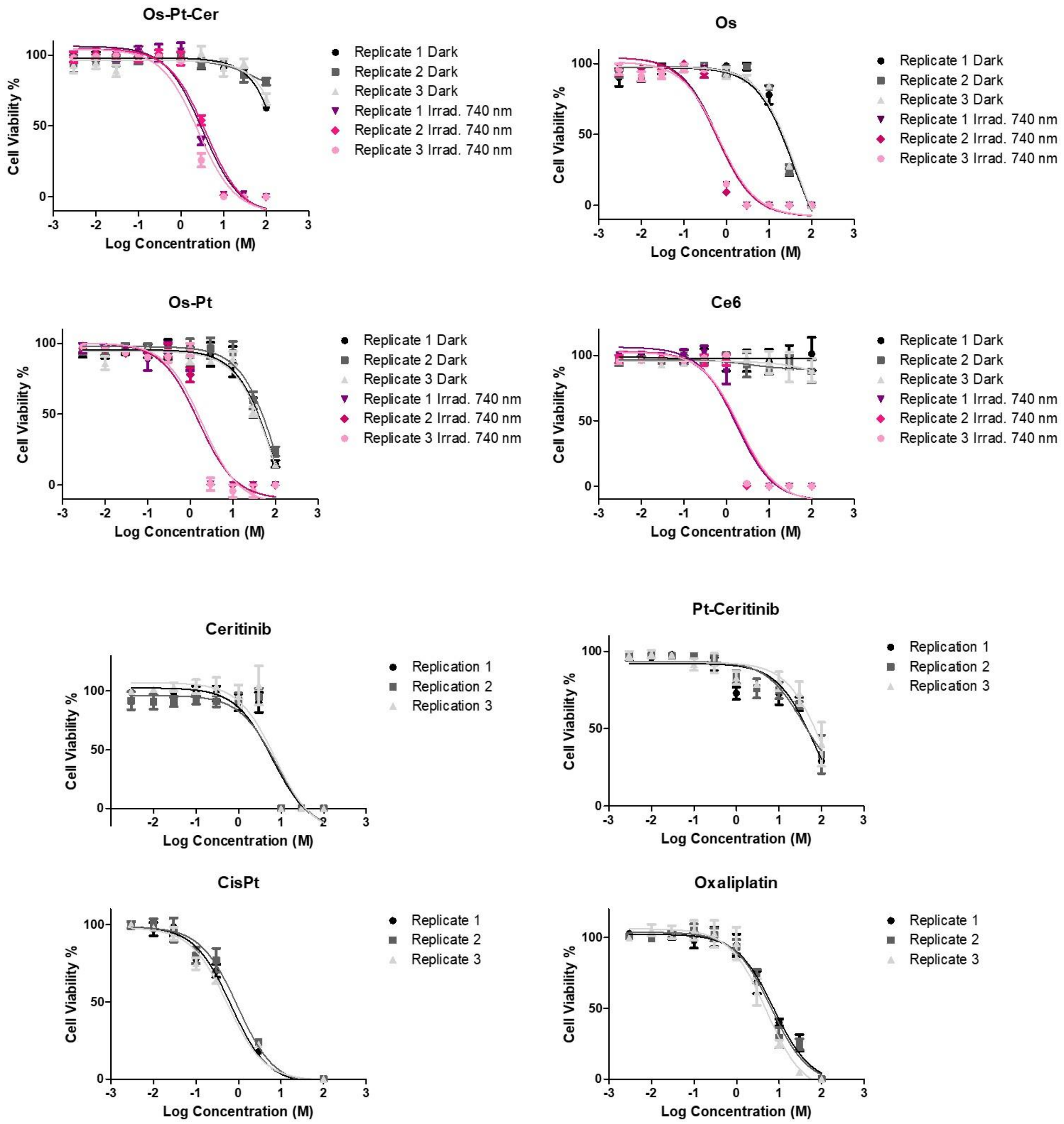

Figure S27. IC_{50} curves of studied compounds were obtained after resazurin cell viability assay in MCA205 cell line at 48 h, in the dark, and irradiated at 740 nm.

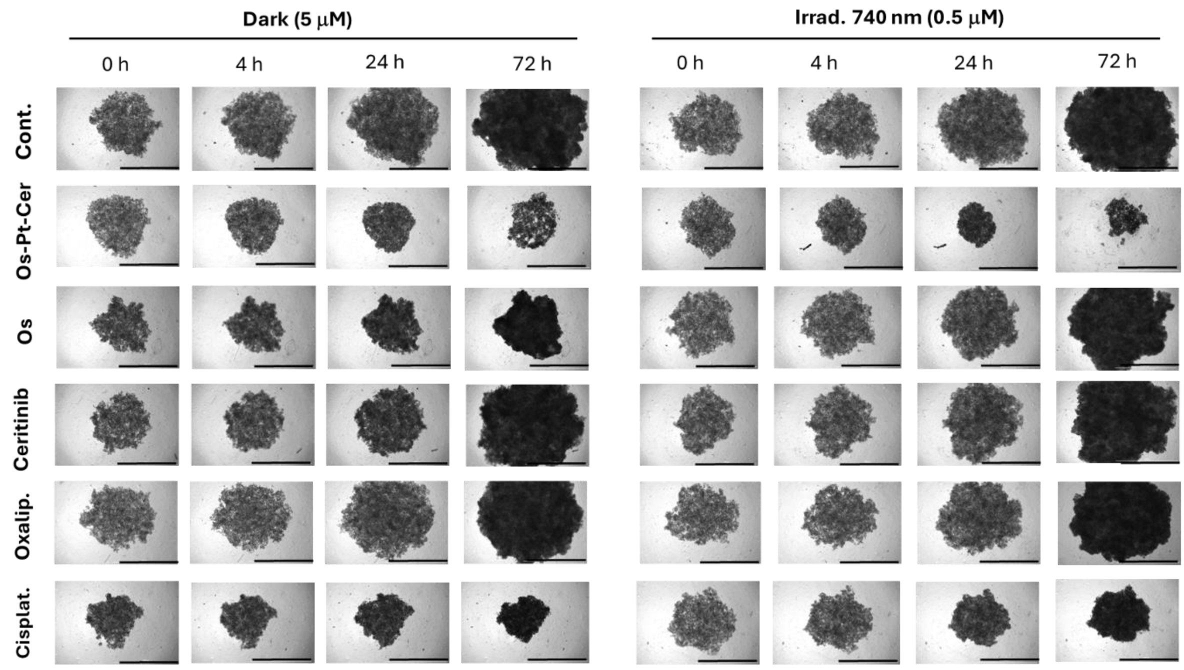

Figure S28. Changes in the growth kinetics of A2780 MCTSs treated with studied compounds at 0.5 and 5 µM for irradiated and dark groups, respectively. Images were collected on 0, 4, 24, and 72 h after the creation of the groups. Scale bar: 1000 µm.

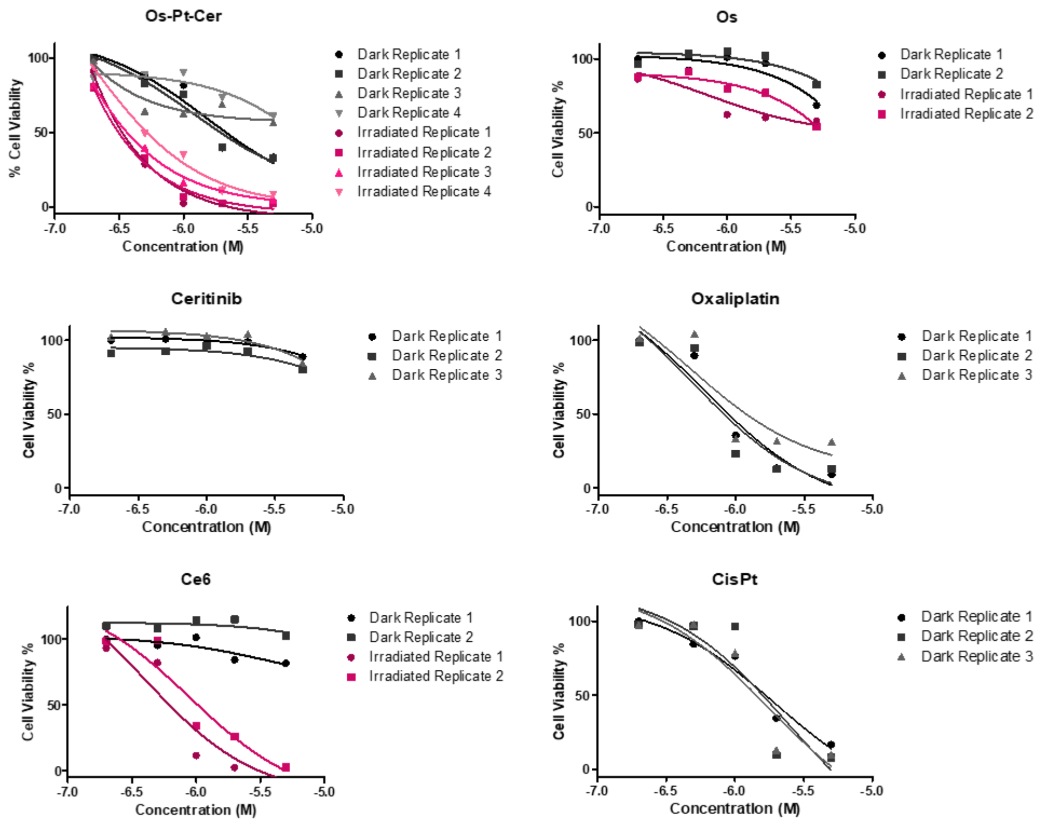

Figure S29. Cell viability graphs after one week of incubation. Studied compounds were incubated for 24 h, thereafter the medium was renovated. For PDT, plates were irradiated at 740 nm for 1h using a LUMOS-BIO photoreactor (Atlas Photonics). For both PDT and regular cytotoxicity, cells were incubated for one week at 37 °C, 5 % CO_2. The medium was renovated each two days. Then, the medium was replaced with 100 µL of fresh medium containing 0.2 mg/mL resazurin (Acros Organics). After 24 h of incubation, the fluorescence signal of the resazurin product was recorded (λexc = 540 nm; λem = 590 nm) using a Cytation 5 Cell Imaging Multi-Mode Reader (Biotek - Agilent).

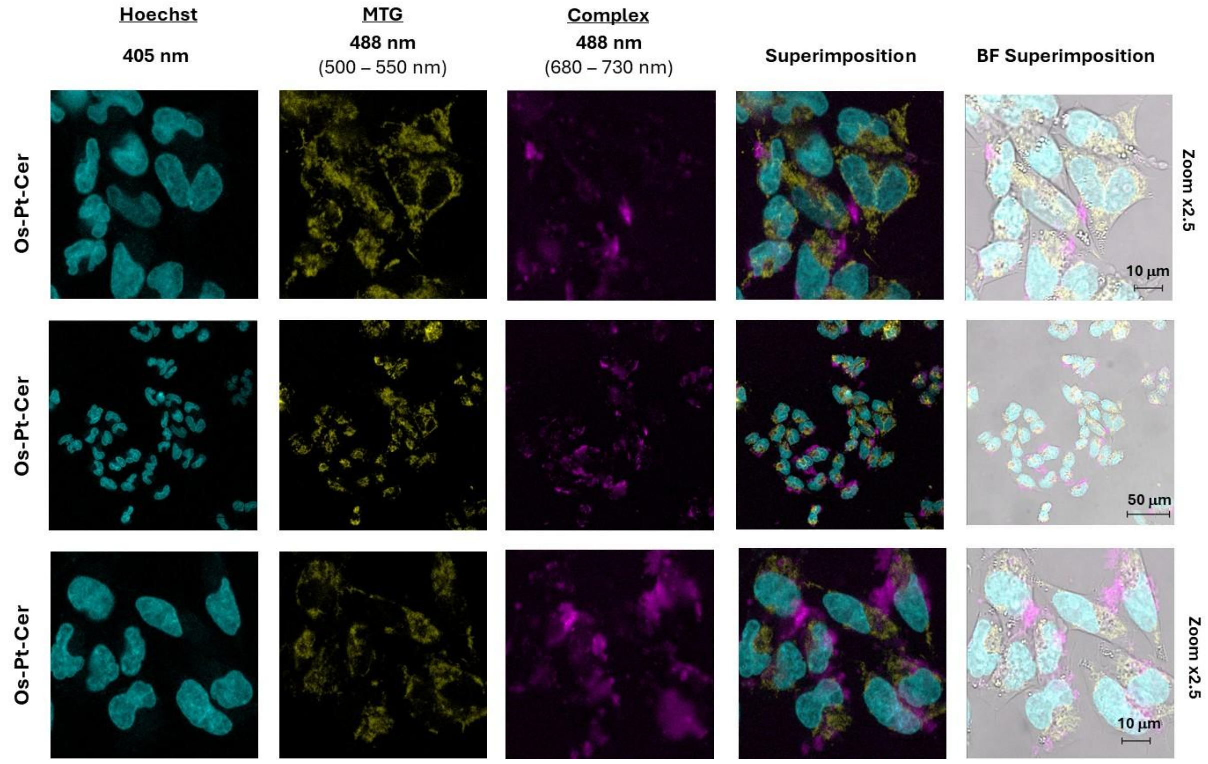

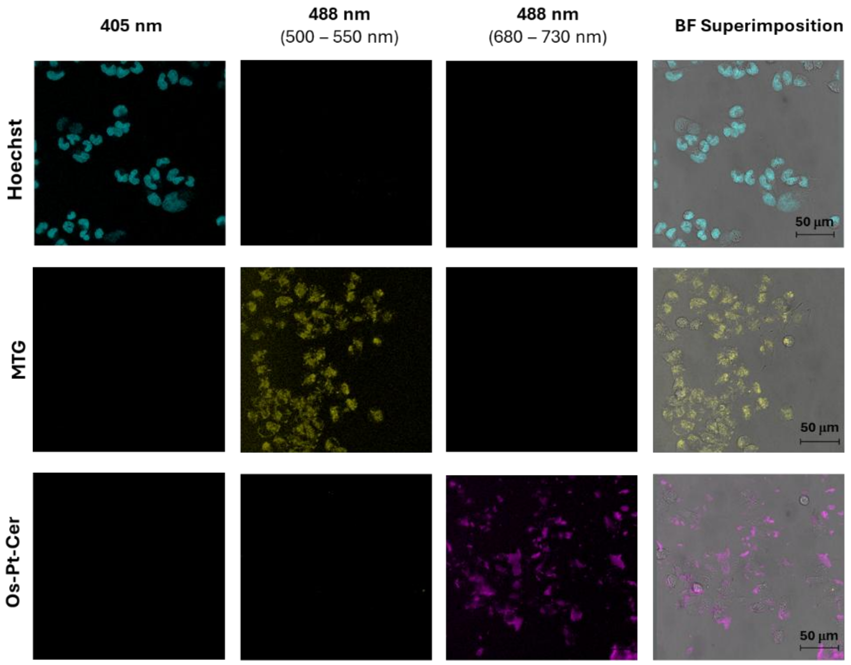

Figure S30. Fluorescence confocal microscopy images of A2780 cells incubated with **Os-Pt-Cer** and stained with MTG and Hoechst. Images recorded after λ_{irra}= 405 nm to visualize complex Hoechst; λ_{irra} = 488 nm (detection window between 500 and 550 nm) to visualize MTG; λ_{irra} = 488 nm (detection window between 680 and 730 nm). Scale bar 50 µM.

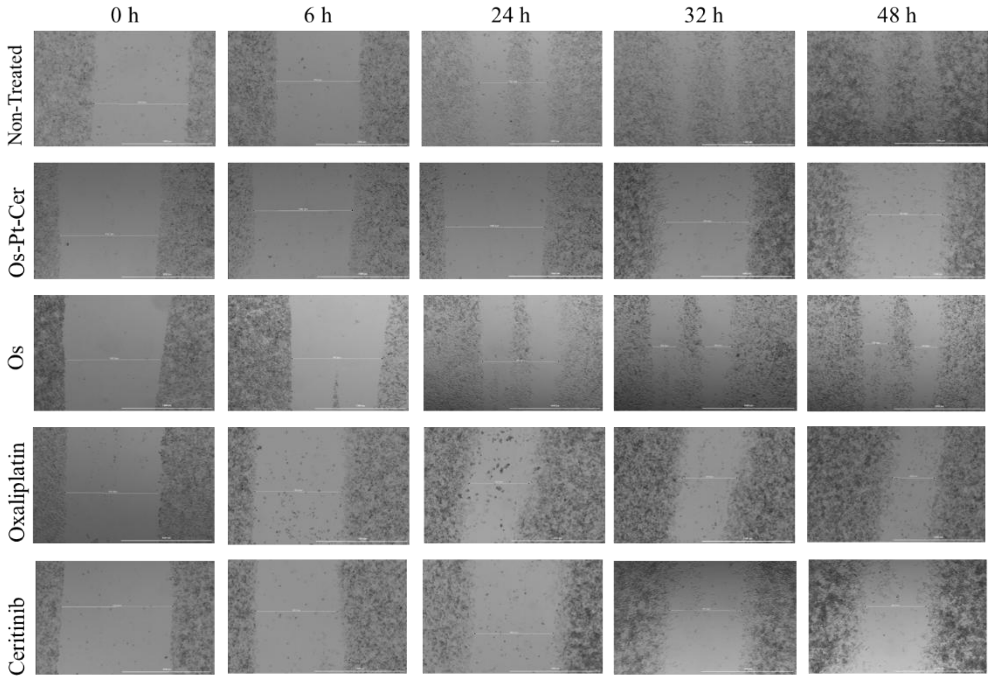

Figure S31. Migration images of A2780 cells after 0, 6, 24, 32 and 48 h of treatment with IC_{20} concentrations of **Os-Pt-Cer**, **Os**, Oxaliplatin and Ceritinib, respectively, or non-treated cells. The images are representative of one successive experiment.

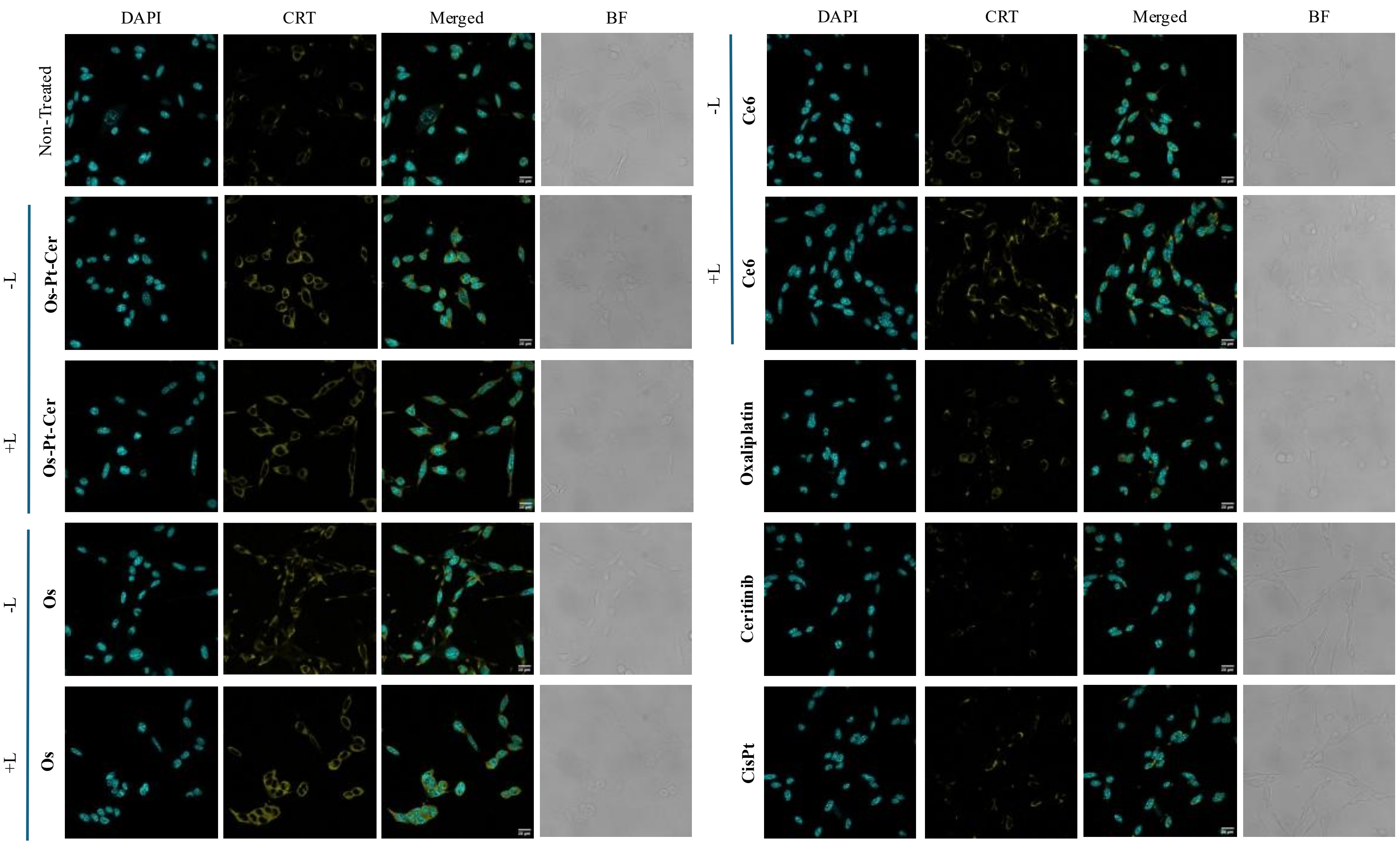

Figure S32. Translocation and changes in calreticulin (CRT) expression on MCA205 cells determined by confocal fluorescence microscopy after 4 h treatment under dark (-L) and light (+L) (740 nm, 1 h) conditions, and an additional 16 h of incubation.

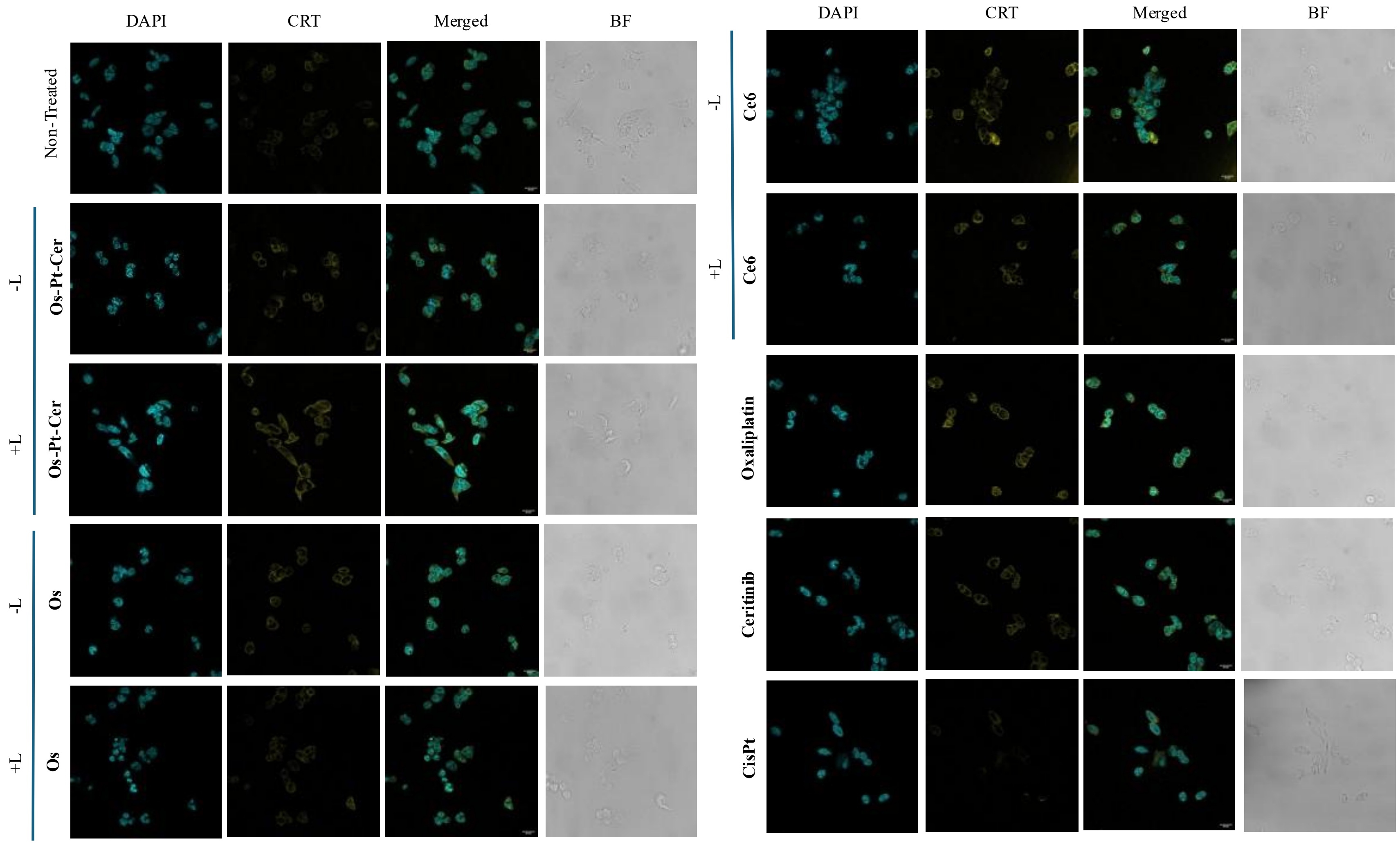

Figure S33. Translocation and changes in calreticulin (CRT) expression on A2780 cells determined by confocal fluorescence microscopy after 4 h treatment under dark (-L) and light (+L) (740 nm, 1 h) conditions, and an additional 16 h of incubation.

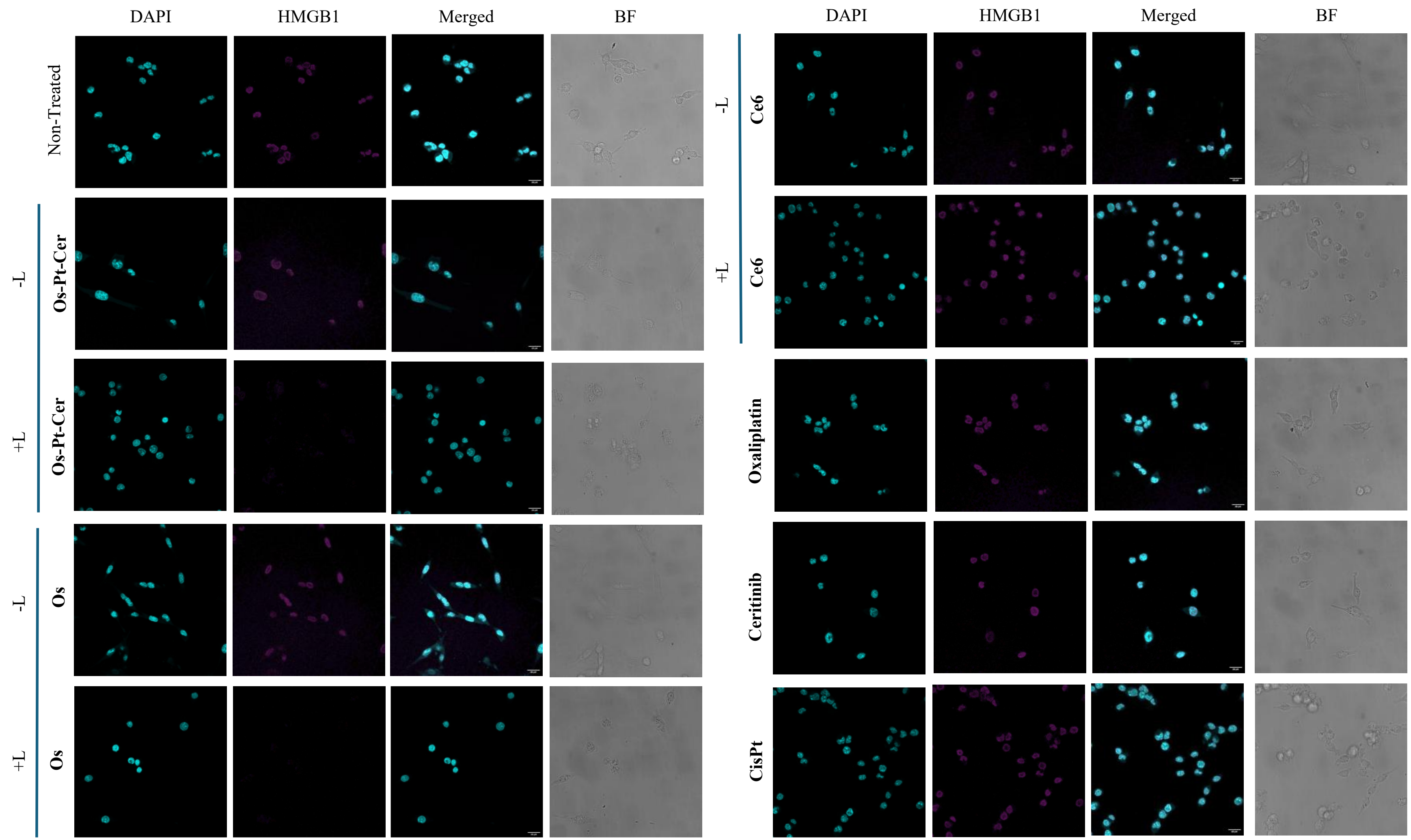

Figure S34. Release of HMGB1 measured by confocal microscopy in MCA205 cells after 4 hours of incubation under dark (-L) and light (+L) (740 nm, 1 h).

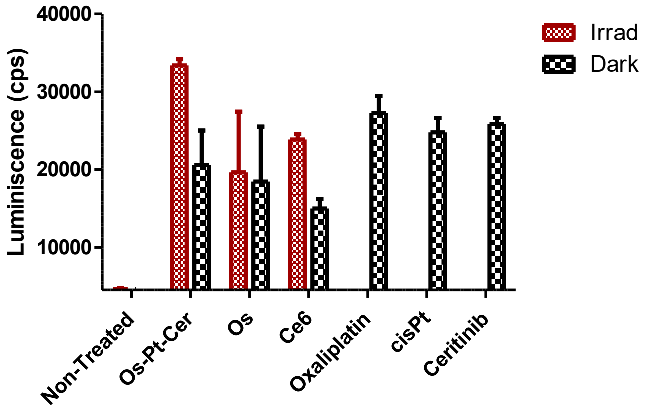

Figure S35. Quantification of extracellular ATP levels using a luciferase-based assay (ab113849) following 4 h incubation in A2780 cells.
